# Development and Spatial External Validation of a Predictive Model of Survival Based on Random Survival Forest Analysis for People Living With HIV and AIDS After Highly Active Antiretroviral Therapy in China: Retrospective Cohort Study

**DOI:** 10.2196/71257

**Published:** 2025-06-02

**Authors:** Xiaoshan Li, Yanhui Li, Zhengping Zhu, Bingxin Tan, Xiaoyi Zhou, Hongjie Shi, Xin Li, Ping Zhu, Yuanyuan Xu

**Affiliations:** 1 Department of Lung Transplantation Center Wuxi People's Hospital Wuxi China; 2 School of Public Health Nantong University Nantong China; 3 Department of AIDS/STD Control and Prevention Nanjing Municipal Center for Disease Control And Prevention Nanjing China; 4 Department of AIDS/STD Control and Prevention Nantong Municipal Center for Disease Control and Prevention Nantong China

**Keywords:** people living with HIV and AIDS, prognostic model, spatial external validation, machine learning, random survival forest

## Abstract

**Background:**

HIV infection remains a global public health challenge, with an estimated 42.3 million cumulative deaths to date. Given the heterogeneity among people living with HIV and AIDS, there is a critical need to develop robust prognostic models to predict survival and guide individualized clinical management.

**Objective:**

We aimed to develop and externally validate a predictive model for the survival of people living with HIV and AIDS following the initiation of highly active antiretroviral therapy (HAART) in China.

**Methods:**

We used data from the HIV and AIDS epidemic surveillance system of the National Center for AIDS/STD Control and Prevention, Chinese Center for Disease Control and Prevention, for this retrospective cohort study. The training set and the external validation set included people living with HIV and AIDS from the cities of Nanjing and Nantong, respectively. The prediction model was developed by using the random survival forest (RSF), and its performance was evaluated against the Cox model, integrated area under the curve (iAUC), consistency index (C index), calibration curves, integrated Brier score (iBS), and decision curve analysis.

**Results:**

A total of 8960 patients were eligible for this study, consisting of 5261 (58.71%) cases in the training set (mean age 32.39, SD 13.30 years; n=4891, 92.97% male patients) and 3699 (41.28%) cases in the external validation set (mean age 43.31, SD 14.18 years; n=3086, 83.42% male patients). The RSF model was developed based on the top 7 variables ranked by variable importance, including hemoglobin, age at HAART treatment, infection route, white blood cell count, education level, blood glucose, and the CD4 count before HAART. The RSF model exhibited good performance, with an iBS of 0.129 in the internal validation set and 0.113 in the external validation set, and a C index of 0.896 (95% CI 0.885-0.906) in the internal validation set and 0.756 (95% CI 0.730-0.782) in the external validation set, respectively. The iAUC was 0.917 (95% CI 0.906-0.929) for the internal validation set and 0.750 (95% CI 0.724-0.776) for the external validation set. Using the Cox model as the benchmark model, the variables included in the RSF model yielded an iBS of 0.172 and 0.115, a C index of 0.829 (95% CI 0.815-0.842) and 0.742 (95% CI 0.714-0.770), and an iAUC of 0.871 (95% CI 0.856-0.885) and 0.740 (95% CI 0.711-0.768) for the internal and external validation sets, respectively.

**Conclusions:**

A machine learning–based RSF model demonstrated promising potential for providing personalized and accurate survival predictions and effective prognostic stratification for people living with HIV and AIDS following HAART in China. Compared to the Cox model, the RSF model exhibited slightly superior performance. A web-based application of the RSF model provides a practical tool for risk assessment and clinical decision-making.

## Introduction

### Background

HIV infection and AIDS are major global public health problems that have claimed about 40.4 million lives worldwide so far [1]. With the successful implementation of highly active antiretroviral therapy (HAART), the life expectancy of people living with HIV and AIDS has improved to a level comparable to the general population. However, due to compromised immune systems, people living with HIV and AIDS still experience secondary infections or cancer, substantially increasing their mortality risk [2], and this underscores the necessity of a prediction model for the survival outcomes of people living with HIV and AIDS.

The established survival and prognosis model for people living with HIV and AIDS revealed that demographic factors, such as age, sex, and BMI, as well as laboratory indicators, such as the World Health Organization (WHO) clinical staging of HIV and AIDS, CD4 T lymphocyte count (CD4), viral load, and hemoglobin, significantly impacted the survival and prognosis of people living with HIV and AIDS [3-14]. However, most previous prediction models were based on traditional univariate and multivariate analyses, which were limited by the inability to address multicollinearity [4,5]. The random survival forest (RSF) is an advanced machine learning algorithm that has been increasingly applied in survival data analysis. In contrast to the conventional Cox proportional hazards model, the RSF is not constrained by multicollinearity among variables, thereby eliminating the need for related assumptions. Despite its ensemble tree-based structure and inherent “black-box” nature, which precludes the direct estimation of regression coefficients, the RSF enables a quantitative assessment of each predictor’s contribution to the outcome via metrics such as variable importance (VIMP) and Shapley additive explanations values [15]. By incorporating bootstrap aggregating (bagging) and introducing randomness in both sample selection and feature subsets, the RSF enhances robustness to noise and outliers, thereby improving model stability and resilience [16]. These advantages make the RSF a powerful and reliable tool for analyzing complex survival data [17]. To our knowledge, only one study has used the RSF method to establish prognostic models for the survival of people living with HIV and AIDS in China [18], with good internal performance (area under the curve [AUC] >0.7); however, the models were not externally validated.

Performance evaluation of prediction models should not be limited to the initial dataset (ie, training set) but should be extended to the external datasets, which can address potential performance disparities arising from the differences in demographics, clinical indicators, medication regimens, and survival outcomes across different datasets or populations [19]. Thorough evaluation in new, spatially distinct data is essential to ascertain the models’ effectiveness and suitability for clinical use. In China, some multivariate prognosis models for people living with HIV and AIDS were established and demonstrated good performance [4]; however, due to the lack of external validation, their applicability in diverse clinical settings was limited.

### Objectives

In this study, we aimed to develop a survival prediction model tailored to Chinese people living with HIV and AIDS following the initiation of HAART by comparing the predictive performance of models constructed using the RSF algorithm and the Cox model in both the training set (ie, internal validation set) and a spatially distinct external validation cohort.

## Methods

### Ethical Considerations

This study was approved by the Ethics Committee of Wuxi People’s Hospital (KY24074) and registered in the medical research registration information system of the National Health Insurance Information Platform (237920) and the China Clinical Trial Registry (ChiCTR2400091128). Informed consent has been waived by using cases and biological specimens obtained from previous clinical diagnoses and treatments. All personal data in the study have been anonymized during the data organization and statistical analysis stages to ensure participant privacy. No compensation was provided to the participants. Methods and results are reported according to the TRIPOD (Transparent Reporting of a Multivariable Prediction Model for Individual Prognosis or Diagnosis) guidelines (Multimedia Appendix 1).

### Data Source

The HIV and AIDS epidemic surveillance system of the National Center for AIDS/STD Control and Prevention (NCAIDS/STD), Chinese Centers for Disease Control and Prevention (CDC), comprises a comprehensive, nationwide framework for data collection and monitoring, aiming at tracking and analyzing data on people living with HIV and AIDS across China. This system collects demographic, clinical, and laboratory information relevant to HIV and AIDS. The demographic information, clinic characteristics, and routine blood biochemical examinations used in this study are sourced from the NCAIDS/STD system.

### Study Design and Participants

From 2005 to 2022, there were 6564 people living with HIV and AIDS in Nanjing and 6608 people living with HIV and AIDS in Nantong, based on data from the HIV and AIDS epidemic surveillance system of the NCAIDS/STD. Patients were included in the model development and validation if they (1) initiated HAART between January 1, 2005, and December 31, 2022; (2) underwent a complete blood test within 3 months before the initiation of HAART; (3) had at least one follow-up record within 1 year following the initiation of HAART; (4) were 18 years or younger at the time of the initiation of HAART; and (5) resided within the local area (including temporary residents). Patients were excluded if the survival status was unavailable, the follow-up time was insufficient (<1 year), time of the initiation of HAART was missing or unknown, or with implausible survival time (<0 days). Among all eligible patients, people living with HIV and AIDS in Nanjing formed the training set and internal validation set, while those in Nantong constituted the external validation dataset (Multimedia Appendix 2).

### Outcome and Candidate Variables

The outcome of this study was survival time, defined as the time from the date of the initiation of HAART to the date of all-cause deaths or the end of observation (December 31, 2022), whichever came first. Candidate factors were selected based on a combination of literature review and expert consultations (Multimedia Appendix 3). Details on the percentages of missing values for each variable in the training and external validation sets are provided in Multimedia Appendix 4. To ensure data integrity and reliability, we excluded variables with more than 20% of missing values. Finally, 17 candidate variables were included in the analysis, including age, sex, marital status, education level, BMI, infection route, history of sexually transmitted disease, WHO clinical stage, CD4, blood glucose (BG), viral load, white blood cell (WBC), platelet, hemoglobin, serum creatinine, alanine aminotransferase, total bilirubin, and HAART regimen.

### Sample Size

The sample size was estimated based on a 4-step procedure using the “pmsampsize” package in R (R Foundation for Statistical Computing) software (equation 1) [20]. A minimum sample size of 1501 was required for our study, assuming an *R*^2^ value of 0.15 (R^2^_CS_), the number of events with candidate predictive parameters of 24 (*P*) and the risk of all-cause death in the population of 2% (*δ*max). Given the substantially lower number of all-cause deaths compared to the number of nonevents, a combination of oversampling and undersampling techniques was used to achieve data balance. To adjust for the data sampling, the ratio of all-cause deaths to nonevents was set at 1:3. On the basis of this ratio, the total sample size for the training set was determined to be 2000 individuals:

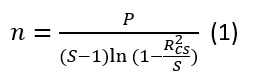



among which:

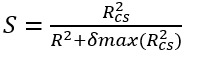



### Statistical Analysis

Data analysis was performed in R (version 4.3.2; R Foundation for Statistical Computing). Continuous variables with a normal distribution were described by mean and SD and compared between the training and external validation sets using independent sample *t* tests. Continuous variables that did not follow a normal distribution were described by the median and IQR and compared between groups by Mann-Whitney *U* tests. Categorical variables were presented by frequencies and percentages and were compared between groups by Pearson chi-square tests. All tests were 2-sided tests, and *P*<.05 was considered statistically significant, .5≤*P*<1 was considered marginally statistically significant [21].

### Data Preprocessing

In the training set (ie, internal validation set), all original variables were first categorized as either categorical or continuous to meet the requirements for subsequent modeling and analysis. For variables with less than 20% missing data, imputation was performed using the “randomForestSRC” package, which is based on the random forest framework and uses a single-iteration strategy. Under a fixed random seed, this method iteratively refines the imputation results to generate a complete dataset. Following imputation, data balancing was conducted using the “ROSE” package, which applies a hybrid technique combining oversampling and undersampling to mitigate the impact of class imbalance on model performance. As the external validation set was used to evaluate the model’s predictive performance and generalizability in real-world clinical scenarios, we did not apply resampling techniques. The processed datasets were then used for subsequent model development and performance evaluation.

### Development of the Prediction Models

**Figure 1:**
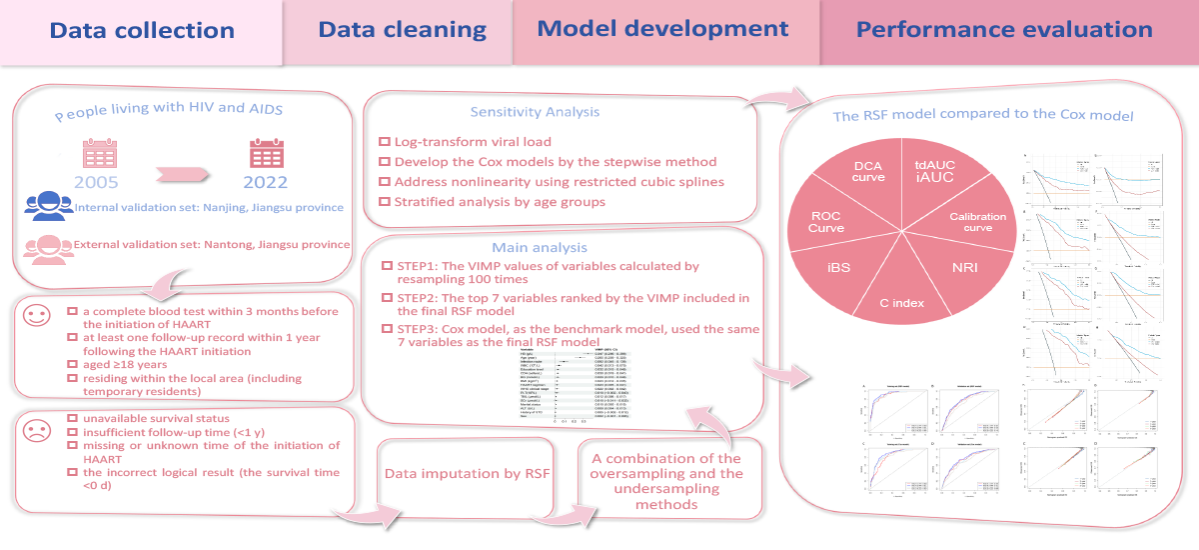
Flowchart of model development. C index: consistency index; Cox: Cox proportional hazards; DCA: decision curve analysis; HAART: highly active antiretroviral therapy; iAUC: integral area under the curve; iBS: integrated Brier score; NRI: net reclassification improvement; ROC: receiver operating characteristic; RSF: random survival forest; tdAUC: time-dependent area under the curve; VIMP: variable importance.

The “randomForestSRC” package was used to construct the RSF model. All candidate variables were included to construct a full-variable RSF model. As the RSF model does not generate regression coefficients for direct results interpretation, the importance of each variable was assessed using the VIMP method, which involved applying out-of-bag data into the survival tree, allowing for random assignment to any child node while calculating the new total cumulative risk. Higher VIMP indicates higher importance of the candidate variables [22]. To enhance accuracy, the self-lifting sampling method was used with 100 repetitions, and 95% CIs for VIMP were calculated. In the main analysis, all variables with a statistically significant VIMP in the full-variable RSF model were initially included. Subsequently, the least important variable was removed sequentially until a decline in model performance was observed. The most parsimonious model was then designated as the final RSF model. Hyperparameter tuning was conducted using the grid search method to optimize model performance [23]. The Cox proportional hazard model was developed using the same variables as the final RSF model using the “Survival” package. The construction process of the model is shown in Figure 1.

### Comparison of the Prediction Models

Net reclassification improvement (NRI) value was used to compare the performance of the RSF and the Cox models, which measured the improvement of the RSF model relative to the Cox model (ie, benchmark model), with a positive NRI indicating better performance of the RSF model. The performance of the RSF model was compared with that of the Cox model using the DeLong test for the AUC of receiver operating characteristic (ROC) and the McNemar test, which incorporates measures such as sensitivity and specificity.

### Evaluation and Validation of the Prediction Models

Internal validation was performed in the training set (ie, internal validation set) using the bootstrap method with 1000 iterations, while external validation was performed using a spatially independent external cohort. Both internal and spatial external validation were evaluated using discrimination, calibration, and clinical applicability. Discrimination indicators include ROC curves, integrated AUC (iAUC), and time-dependent AUC (tdAUC). Calibration indicators include a consistency index (C index), calibration curve, and integrated Brier score (iBS). Clinical applicability indicators include decision curve analysis (DCA) and risk stratification.

ROC curves were plotted at different time points. An iAUC or tdAUC of <0.5, 0.5 to 0.7, and 0.7 indicated no, average, and good predictive capability, respectively. The calibration curves were used to assess the consistency of the predicted values and the observed values. The C index was used to represent the degree of discrimination, with values of >0.9, 0.7 to 0.9, and 0.5 to 0.7 indicating high, moderate, and low discrimination, respectively [24]. To determine calibration accuracy, we evaluated the closeness of the calibration curve to the ideal straight line (represented as y=x), where a closer proximity signifies higher calibration accuracy. The iBS measured the closeness between the model’s predicted risks and the observed probabilities, with lower scores indicating better performance [25]. DCA curves were used to evaluate the clinical benefits of the model, namely its practical utility in influencing clinical decisions. A higher net benefit at a given threshold indicated greater patient benefit from using the model for diagnostic purposes.

### Risk Stratification

To guide clinical decision-making and enable precise diagnosis and treatment, the final RSF model was used for risk stratification. Using X-tile software (Yale University), individuals were categorized into 3 risk groups—low risk (better survival time), intermediate risk (average survival time), and high risk (poorer survival time)—or 2 groups—low risk (better survival time) and high risk (poorer survival time). Kaplan-Meier survival curves were plotted based on the risk scores predicted by the RSF model, and the differences in survival times among these groups were evaluated. Furthermore, survival analysis and Cox model were used to assess the impact of risk groups on patient prognosis. To quantify the survival status across various risk strata, we calculated the survival probabilities and CIs at specific time points. In addition, the mean survival times and CIs were derived to comprehensively evaluate the survival outcomes among the different risk groups.

### Sensitivity Analyses

In total, 4 sensitivity analyses were performed. First, previous studies [6,8] identified viral load as a crucial predictor. However, due to the high proportion of missing values (2566/5261, 48.7% and 1403/3699, 37.9% in the training and external validation sets, respectively) in this study, it was not included in the main analysis. In the sensitivity analysis, we included viral load in the RSF model, and its performance was reevaluated by comparing the NRI values with the original final RSF model. Second, Cox models were developed using a stepwise method and their performance was compared with the original final RSF model. Third, restricted cubic splines were used to address the nonlinear relationships between the continuous variables (serum creatinine, BG, and CD4) and the survival rates in the RSF models and the Cox models. Finally, stratified analysis by age groups (<60 years and ≥60 years) was performed to evaluate the performance of the RSF models in different age groups.

## Results

### Participant Characteristics

This study included 5261 (N=8960, 58.71%) eligible patients in the training set (mean age 36.5, SD 13.2 years; n=4891, 93% male patients) and 3699 (41.28%) patients in the external validation set (mean age 44.1, SD 14.2 years; n=3086, 83.4% male patients). Table 1 shows the differences in the demographic and clinical characteristics of patients between the training set and the external validation set. All variables, except for WBC, were different between the training set and external validation set. In the training set, the 1-, 3-, 5-, and 8-year survival rates were 98.6% (95% CI 92.7%-99.1%), 97.5% (95% CI 90.7%-97.7%), 96.9% (95% CI 94.2%-97.5%), and 96.3% (95% CI 95.6%-96.9%), respectively. In the validation set, the corresponding survival rates were 96.5% (95% CI 95.9%-97.1%), 94.3% (95% CI 93.5%-95.1%), 92.4% (95% CI 91.4%-93.3%), and 90.5% (95% CI 89.3%-91.8%).

**Table 1:** Demographic and clinical characteristics of patients in the training set and the external validation set.

Variables					Total set (N=8960)			Training set (n=5261)			External validation set (n=3699)			*t* test (*df*)				*P* value				Chi-square (*df*)				*z* score	
**Demographic characteristics**																											
	Age (y), mean (SD)			39.7 (14.1)			36.5 (13.2)			44.1 (14.2)			—				<.001				—				−25.755		
	**Sex,** **n (%)**														—				<.001				202.3 (1)				—
		Male	7977 (89)			4891 (93)			3086 (83.4)																		
		Female	983 (11)			370 (7)			613 (16.6)																		
	**Marital status,** **n (%)**														—				<.001				907.0 (1)				—
		Unmarried	3914 (43.9)			2991 (57.1)			923 (25)																		
		Married	5011 (56.1)			2244 (42.9)			2767 (75)																		
	**Education level,** **n (%)**														—				<.001				—				35.105
		Illiterate or primary school	917 (10.2)			324 (6.2)			593 (16)																		
		Middle school	2353 (26.3)			883 (16.8)			1470 (39.7)																		
		High school	2041 (22.8)			1173 (22.3)			868 (23.5)																		
		College and above	3649 (40.7)			2881 (54.8)			768 (20.8)																		
	**BMI (kg/m^2^), n (%)**														—				.07				—				1.806
		<18.5	906 (10.1)			535 (10.2)			371 (10)																		
		18.5-23.9	5895 (65.8)			3501 (66.5)			2394 (64.7)																		
		＞23.9	2159 (24.1)			1225 (23.3)			934 (25.3)																		
**Clinical characteristics**																											
	**Infection route, n (%)**														—				<.001				—				−17.298
		Homosexual transmission	5636 (63.1)			3719 (71)			1917 (52)																		
		Heterosexual transmission	3156 (35.3)			1406 (26.8)			1750 (47.5)																		
		Other	136 (1.5)			117 (2.2)			19 (0.5)																		
	**History of STD^a^, n (%)**														—				.002				10.1 (1)				—
		No	6174 (79.6)			3329 (78.3)			2845 (81.2)																		
		Yes	1583 (20.4)			924 (21.7)			659 (18.8)																		
	**WHO^b^ clinical stage, n (%)**														—				<.001				—				11.218
		Stage 1	2477 (27.7)			889 (16.9)			1588 (42.9)																		
		Stage 2	2653 (29.6)			2130 (40.5)			523 (14.1)																		
		Stage 3	2092 (23.4)			1287 (24.5)			805 (21.8)																		
		Stage 4	1736 (19.4)			953 (18.1)			783 (21.2)																		
**Biochemical index**																											
	CD4^c^ (cells/μL), median (IQR)			290.0 (168.0-423.0)			314.0 (196.0-447.0)			263.0 (137.0-395.2)			—				<.001				—				−11.518		
	BG^d^ (mmol/L), mean (SD)			5.7 (1.6)			5.6 (1.4)			6.0 (1.8)			10.368 (8958)				<.001				—				—		
	WBC^e^ (10^9^/L), mean (SD)			5.6 (13.2)			5.7 (2.0)			5.5 (20.4)			—				.7				—				−0.382		
	PLT^f^ (10^9^/L), median (IQR)			188.0 (151.0-230.0)			194.0 (158.0 -234.0)			180.0 (144.0 -222.0)			—				<.001				—				9.520		
	HB^g^ (g/L), mean (SD*)*			142.3 (157.1)			146.8 (204.6)			135.9 (22.8)			—				<.001				—				3.728		
	SCr^h^ (μmol/L), mean (SD)			69.1 (20.4)			71.6 (19.3)			65.6 (21.5)			12.772 (8958)				<.001				—				—		
	ALT^i^ (U/L), median (IQR)			22.9 (15.8-35.9)			22.2 (15.5-35.0)			24.0 (16.0-37.0)			—				<.001				—				−2.646		
	TBIL^j^ (µmol/L), mean (SD)			11.7 (8.3)			11.9 (6.4)			11.5 (10.5)			—				.02				—				2.290		
	**HAART^k^ regimen, n (%)**														—				<.001				—				8.345
		NRTIs^l^ or PiIs^m^ or Mix^n^	226 (2.5)			116 (2.2)			110 (3.0)																		
		NNRTIs^o^	7308 (81.6)			4167 (79.2)			3141 (84.9)																		
		INSTls^p^	1426 (15.9)			978 (18.6)			448 (12.1)																		

^a^STD: sexually transmitted disease.

^b^WHO: World Health Organization.

^c^CD4: CD4 T lymphocyte count.

^d^BG: blood glucose.

^e^WBC: white blood cell.

^f^PLT: platelet.

^g^HB: hemoglobin.

^h^SCr: serum creatinine.

^i^ALT: alanine aminotransferase.

^j^TBIL: total bilirubin.

^k^HAART: highly active antiretroviral therapy.

^l^NRTI: nucleotide reverse transcriptase inhibitor.

^m^PiI: protease inhibitor.

^n^Mix: different types of medicine compound preparation.

^o^NNRTI: nonnucleoside reverse transcriptase inhibitor.

^p^INSTl: integrase inhibitor.

### Development of the Prediction Model

**Figure 2:**
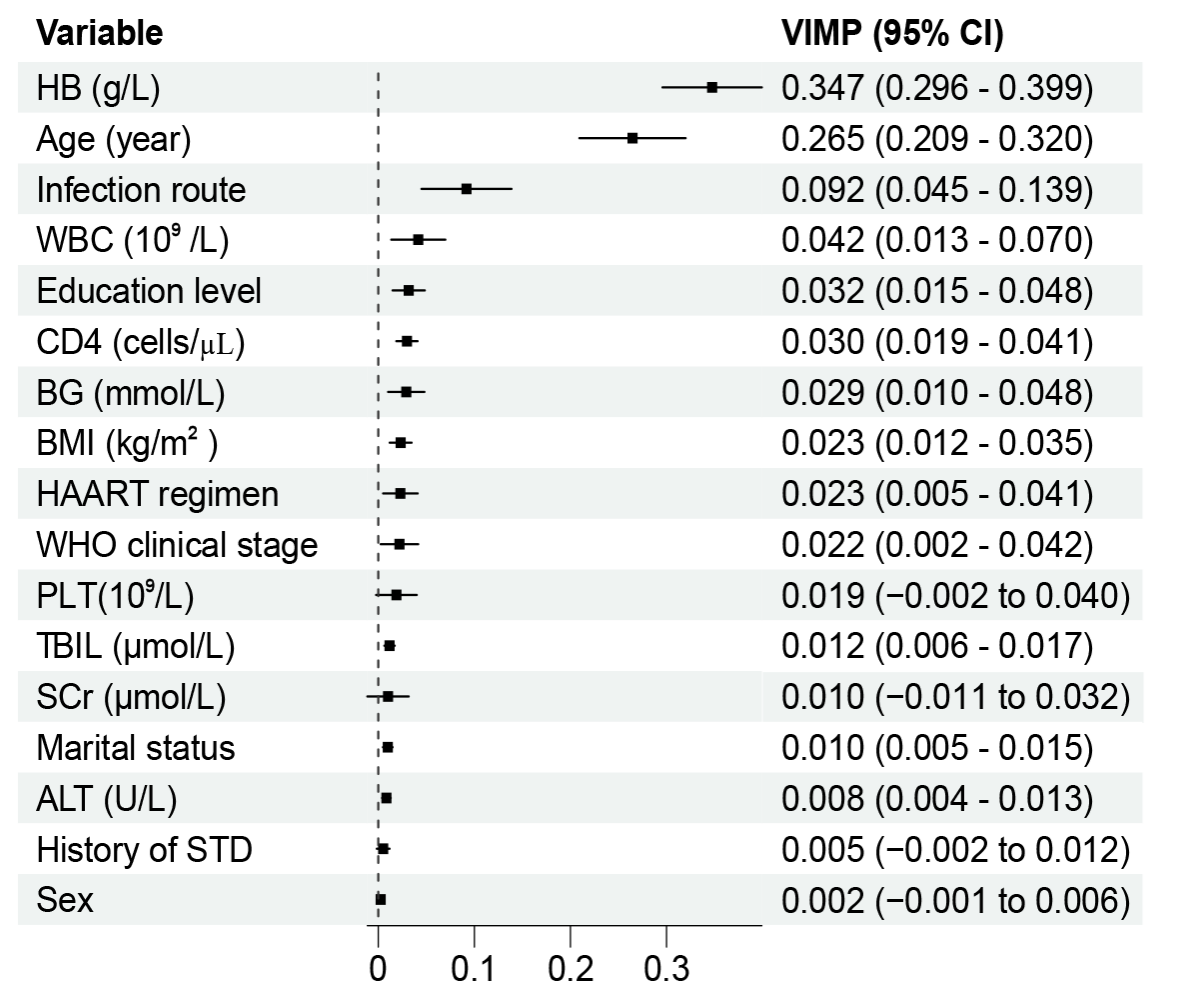
Variable importance (VIMP) plot for each candidate variables in the random survival forest analysis. ALT: alanine aminotransferase; BG: blood glucose; CD4: CD4 T lymphocyte count; HAART: highly active antiretroviral therapy; HB: hemoglobin; PLT: platelet; SCr: serum creatinine; STD: sexually transmitted disease; TBIL: total bilirubin; WBC: white blood cell; WHO: World Health Organization.

Figure 2 shows the VIMP values obtained by 100 repeated self-lifting sampling. The top 7 variables ranked by VIMP were included in the final RSF model: hemoglobin, age, infection route, WBC, education level, CD4, and BG. A web-based tool was developed based on the RSF model [26].

### Evaluation and Validation of the Prediction Models

#### Model Discrimination

**Figure 3:**
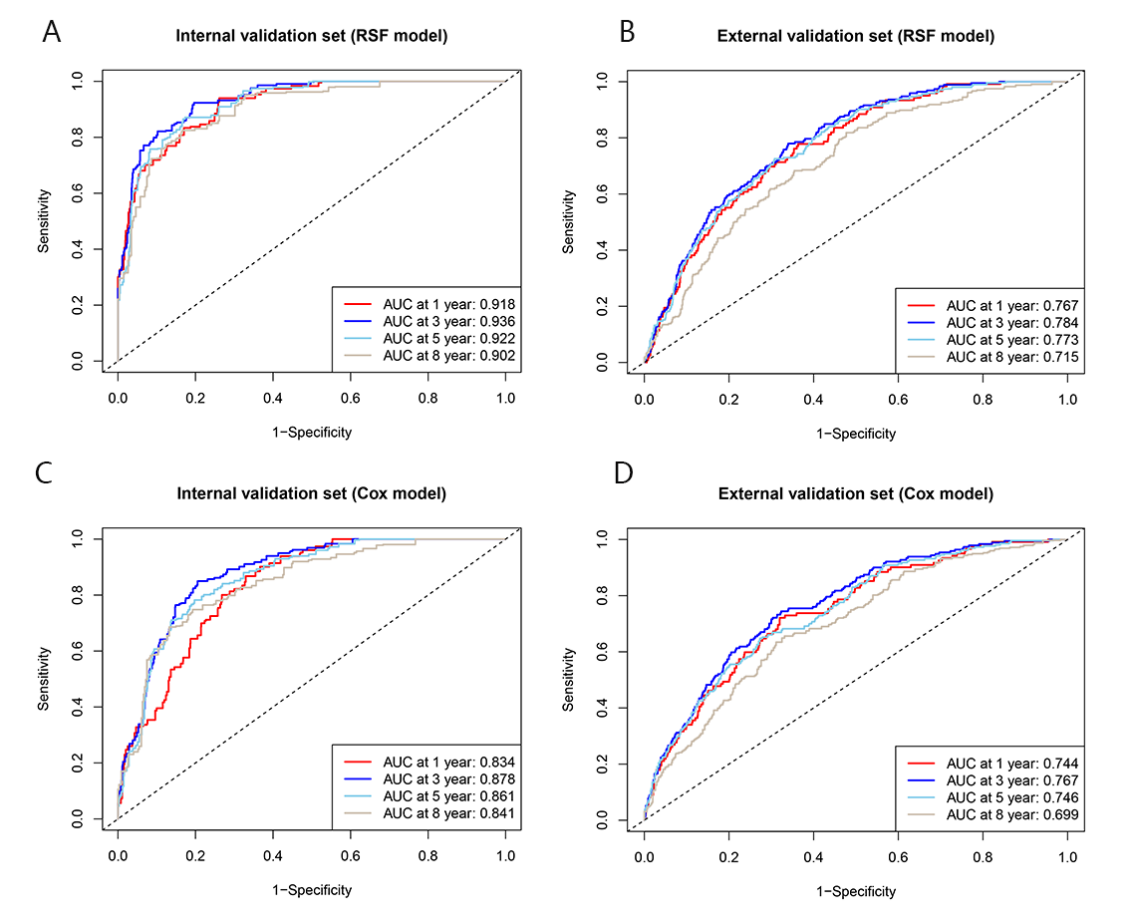
Receiver operating characteristic (ROC) curves of the random survival forest (RSF) model and the Cox proportional hazards (Cox) model. (A) ROC curves of the RSF model in the internal validation set; (B) ROC curves of the RSF model in the external validation set; (C) ROC curves of the Cox model in the internal validation set; and (D) ROC curves of the Cox model in the external validation set. AUC: area under the curve.

Both the RSF model and Cox model demonstrated good predictive capability in the internal validation set, with an iAUC of 0.917 (95% CI 0.906-0.929) in the RSF model and 0.871 (95% CI 0.856-0.885) in the Cox model (Figures 3A and 3C). In the external validation set, the iAUC was 0.750 (95% CI 0.724-0.776) in the RSF model and 0.740 (95% CI 0.711-0.768) in the Cox model (Figures 3B and 3D). The RSF models consistently achieved significantly higher AUC values than the Cox models across all time points in both the internal and external validation sets (Table 2). The DeLong test further provided statistical inference results of the differences in AUC values of the 2 models at various time points (Multimedia Appendix 5). In the internal validation set, the RSF model demonstrated significantly superior discriminative ability at all time points (all *P*<.001). In the external validation set, the differences in AUC values at 1 and 3 years achieved marginal statistical significance (*P*=.05 and *P*=.05, respectively), while the difference at 5 years reached statistical significance (*P*=.003), suggesting that the RSF model also exhibited better predictive performance than the Cox model in the external validation set.

**Table 2:** Time-dependent area under the curve (tdAUC) of the random survival forest (RSF) model and the Cox proportional hazards (Cox) model at various time points following the initiation of highly active antiretroviral therapy.

Time (y)	Internal validation set			External validation set	
	RSF model, tdAUC (95% CI)	Cox model, tdAUC (95% CI)	RSF model, tdAUC (95% CI)		Cox model, tdAUC (95% CI)

1	0.918 (0.902-0.935)	0.834 (0.811-0.856)	0.767 (0.748-0.785)		0.744 (0.721-0.767)
3	0.936 (0.923-0.948)	0.878 (0.859-0.896)	0.784 (0.768-0.799)		0.767 (0.751-0.784)
5	0.922 (0.907-0.937)	0.861 (0.840-0.883)	0.773 (0.754-0.789)		0.746 (0.727-0.765)
8	0.902 (0.880-0.925)	0.841 (0.812-0.871)	0.715 (0.694-0.735)		0.699 (0.675-0.724)

#### Model Calibration

**Figure 4:**
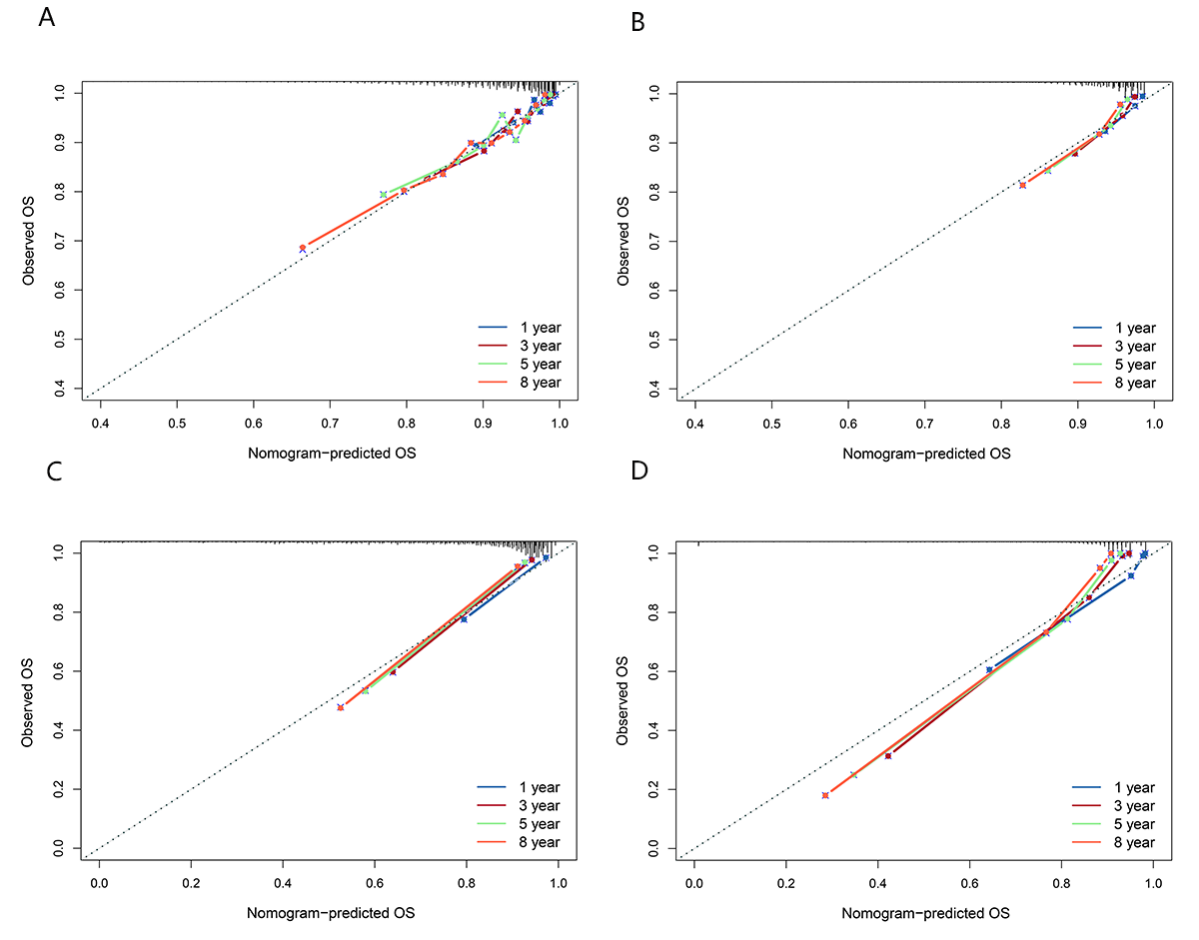
Calibration curves of the random survival forest (RSF) model and the Cox proportional hazards (Cox) model at various time points following the initiation of highly active antiretroviral therapy. (A) Calibration curves of the RSF model in the internal validation set; (B) calibration curves of the RSF model in the external validation set; (C) calibration curves of the Cox model in the internal validation set; and (D) calibration curves of the Cox model in the external validation set. OS: overall survival.

**Figure 5:**
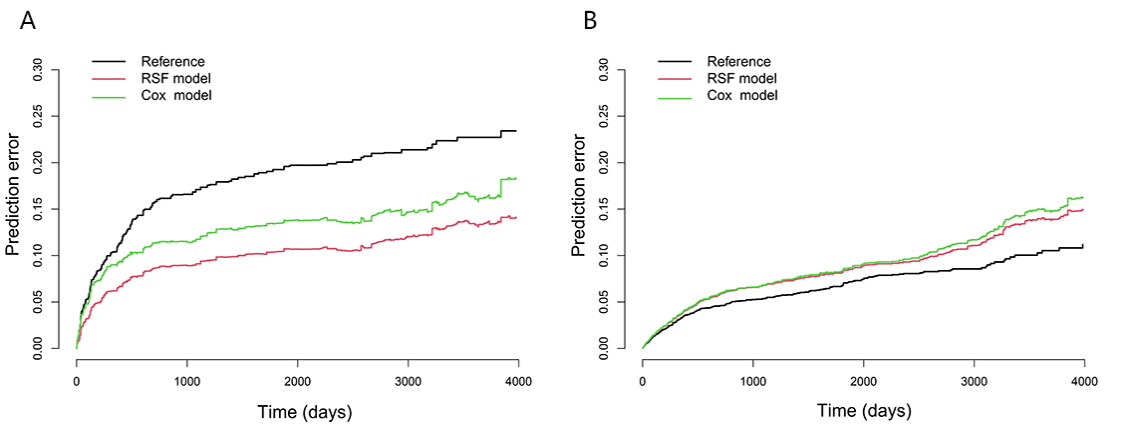
Brier curves of the random survival forest (RSF) model and the Cox proportional hazards (Cox) model. (A) Brier curves of the RSF model and the Cox model in the internal validation set and (B) Brier curves of the RSF model and the Cox model in the external validation set.

The RSF model and the Cox model had high consistency between the predicted and the observed survival rates of people living with HIV and AIDS in both the internal and external validation sets (Figure 4). The C index of the RSF model was 0.896 (95% CI 0.885-0.906) in the internal validation set and 0.756 (95% CI 0.73-0.782) in the external validation set. The C index of the Cox model was 0.829 (95% CI 0.815-0.842) and 0.742 (95% CI 0.714-0.770) in the internal validation set and external validation set, respectively. The iBS of the RSF model in the internal validation set and external validation set were 0.129 and 0.113, respectively, and the iBS of the Cox model in the internal validation set and external validation set were 0.172 and 0.115, respectively (Figure 5).

#### Model Clinical Applicability

**Figure 6:**
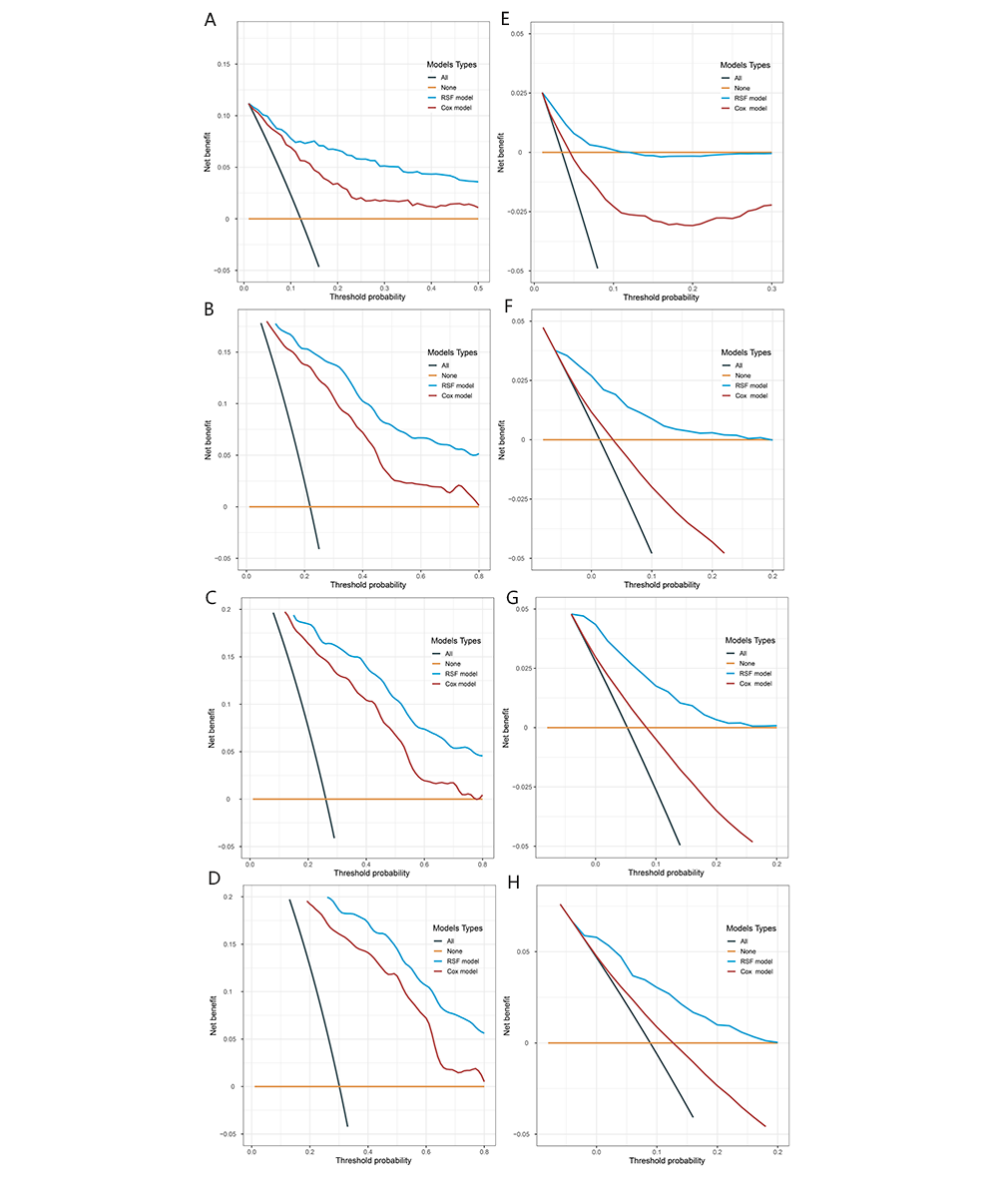
Decision curve analysis (DCA) curves of the random survival forest (RSF) models and the Cox proportional hazards (Cox) models. (A, B, C, and D) DCA curves of the RSF model and the Cox model in the internal validation set and (E, F, G, and H) DCA curves of the RSF model and the Cox model in the external validation set.

The results of the DCA curves showed that the RSF model achieved good clinical benefits in both the internal validation set and the external validation set, and the net benefits of the RSF model were better than the Cox model (Figure 6).

**Figure 7:**
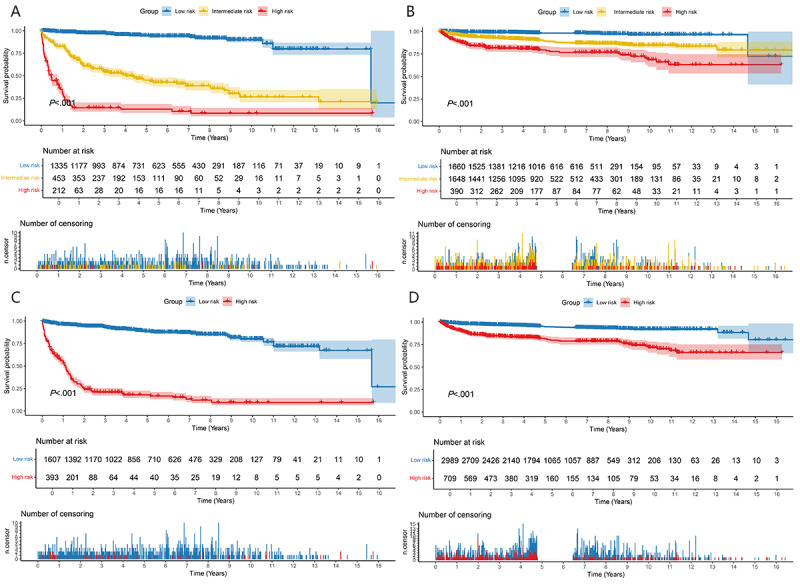
Comparison of survival rates between different risk stratification groups. (A) Low-, intermediate-, and high-risk groups categories in the internal validation set; (B) low-, intermediate-, and high-risk groups in the external validation set; (C) low- and high-risk groups in the internal validation set; and (D) low- and high-risk groups in the external validation set.

The risk stratification analysis of the RSF model revealed statistical differences in overall survival rates among the 3 risk groups in both the internal and external validation sets (Figure 7). Specifically, within the internal validation set, the risk of mortality in the high-risk and intermediate-risk groups was 44.41 times and 11.36 times higher, respectively, compared to the low-risk group (*P*<.001). Similarly, in the external validation set, the mortality risk was elevated by 13.13 times in the high-risk group and 5.65 times in the intermediate-risk group, relative to the low-risk group (*P*<.001). The 1-, 3-, and 5-year survival rates were highest among the low-risk group and lowest among the high-risk group (Multimedia Appendix 6). When risk was dichotomized into 2 groups, the risk of mortality was 14.53 times greater in the high-risk group compared to the low-risk group in the internal validation set and 4.22 times higher in the external validation set (*P*<.001 for both comparisons), respectively.

#### Comparison of the RSF and Cox Models

On the basis of NRI, the RSF model demonstrated superior predictive capacity over the Cox model in the first 8 years post-HAART initiation within the internal validation set (Table 3). However, in the external validation set, the RSF model did not outperform the Cox model in the first 3 years but exhibited advantages over the Cox model after 3 years. We evaluated the sensitivity and specificity of both the RSF and Cox models on both the internal and external validation data (Multimedia Appendix 7). In the internal validation set, the RSF model exhibited superior performance over the Cox model, as evidenced by the McNemar test (*P*<.001 for both), but not observed in the external validation set (Multimedia Appendix 8).

**Table 3:** Net reclassification improvements (NRIs) of the random survival forest (RSF) model compared with the Cox proportional hazards (Cox) model at various time points following the initiation of highly active antiretroviral therapy in the internal and external validation datasets.

Time (y)	Internal validation set, NRI (95% CI)	External validation set, NRI (95% CI)
1	0.386 (0.313-0.463)	−0.007 (–0.068 to 0.002)
2	0.268 (0.166-0.334)	−0.001 (–0.077 to 0.006)
3	0.261 (0.146-0.312)	−0.060 (–0.134 to 0.008)
4	0.223 (0.143-0.291)	0.024 (0.004 to 0.054)
5	0.165 (0.119-0.253)	0.020 (0.004 to 0.060)
6	0.185 (0.116-0.238)	0.040 (0.010 to 0.095)
7	0.181 (0.105-0.235)	0.050 (0.013 to 0.092)
8	0.192 (0.095-0.230)	0.050 (0.016 to 0.111)

### Sensitivity Analysis

The incremental model was built in the first sensitivity analysis. The predictive ability of the incremental RSF model, which included viral load, was lower compared to the original RSF model. The performance of the RSF incremental models did not improve by adding viral load to the RSF model, with or without log transformation (Multimedia Appendix 9).

In the second sensitivity analysis, the RSF model in the main analysis demonstrated superior performance compared to the Cox model developed using a stepwise approach, both in the internal and external validation sets. Specifically, the RSF model exhibited higher discrimination, accuracy, and clinical utility. The improved discrimination indicates that the RSF model was better at distinguishing individuals with higher and lower risks of the outcome. The increased accuracy suggests that the predictions made by the RSF model were closer to the observed outcomes. By calculating the NRI over the first 8 years after HAART initiation, the RSF model consistently outperformed the Cox model in the internal validation set, while in the external validation set, the RSF model surpassed the Cox model starting from the second year (Multimedia Appendix 10). In the third sensitivity analysis, we aimed to ensure the applicability of the Cox model by applying restricted cubic spline transformations to continuous variables that did not exhibit a linear relationship with the survival outcomes. For variables that violated the linearity assumption, we categorized them to meet with the Cox model’s requirements. Through this approach, we reassessed the performance of the RSF model against the Cox model, focusing on discrimination, accuracy, and clinical relevance. Our findings reinforced the superiority of the RSF model for predicting outcomes in people living with HIV and AIDS, as it consistently demonstrated superior discriminative power, a higher degree of accuracy in predictions, and greater clinical utility compared to the Cox model. By calculating the NRI over the first 8 years after HAART initiation, the RSF model consistently outperformed the Cox model in the internal validation set, while in the external validation set, the RSF model surpassed the Cox model starting from the third year. (Multimedia Appendix 11).

In the fourth sensitivity analysis, the generalization ability of the RSF model was tested in different age subgroups. The results showed that RSF had good discrimination in different age groups, particularly in individuals aged <60 years, underscoring its robustness and applicability across diverse age groups. (Multimedia Appendix 12).

## Discussion

### Principal Findings

Although HAART significantly prolonged the life expectancy of people living with HIV and AIDS, opportunistic infections and cancer continue to pose serious threats to their survival [2]. In the past decade, efforts have focused on developing a precise prognosis assessment to optimize clinical decision-making for people living with HIV and AIDS. Nevertheless, conventional approaches based on prognostic factors to identify patient survival outcomes have some limitations, such as insufficient accuracy for a single prognostic factor and difficulty in integrating multiple prognostic factors. The prognostic model, also known as the prediction model, offers an effective solution by generating personalized predictions, addressing these limitations by integrating various factors for individualized survival prediction. For people living with HIV and AIDS, several studies [4,5,27] have presented results of studying prognostic models to predict overall survival or survival at specific time points. However, these models showed limited performance or clinical application, resulting from multicollinearity or lack of external validation. This study developed an externally validated RSF model using data from the HIV and AIDS epidemic surveillance system of NCAIDS/STD, providing a personalized and accurate survival prediction for people living with HIV and AIDS after HAART. We identified 7 critical factors in predicting all-cause deaths among 17 clinical characteristics. In addition, the performance of the RSF model was slightly superior to that of the conventional Cox regression model regarding discrimination, calibration, and clinical applicability. Finally, the RSF model exhibited excellent performance in predicting long-term survival and survival outcomes in older population.

In this study, 7 key variables, including hemoglobin, age at HAART treatment, infection route, WBC count, education level, BG, and CD4 count before HAART, were identified as critical factors predicting all-cause mortality after HAART in people living with HIV and AIDS. Variables such as hemoglobin and age at HAART treatment have also been recognized in previous research as independent predictors highly correlated with mortality after HAART in people living with HIV and AIDS. A meta-analysis [28] reported the association between hemoglobin levels and life span in patients with HIV, highlighting the need for interventions in patients with anemia to improve life expectancy and quality. In a comprehensive review [29], age was found to be inversely correlated with survival time. A study [30] has indicated that among patients who tested positive for HIV, lower levels of education were associated with higher all-cause mortality rates during HAART. Disruption of BG homeostasis is also a crucial factor influencing the survival of patients with AIDS. While HAART has reduced the mortality rate among individuals with HIV, it was associated with increased prevalence of dyslipidemia among patients with AIDS [31], thereby increasing all-cause mortality through an alternative pathway. CD4 cell count is closely associated with the survival rate of patients with AIDS. Generally, a lower CD4 cell count indicates a higher risk of all-cause mortality. More importantly, our findings substantially confirmed the prognostic significance of these variables for survival outcomes using VIMP.

In recent years, various machine learning algorithms have emerged to handle right-censored data in survival analysis, such as deep survival forests and survival support vector machines. These approaches effectively integrate traditional machine learning frameworks with principles of survival analysis, allowing them to manage incomplete observations. Among these, RSF, a classical ensemble method based on bootstrap aggregating, has demonstrated strong predictive performance and model robustness, owing to its dual-layer randomization at both the sample and feature levels. In this study, RSF was selected as the primary modeling approach to explore its potential in prediction for survival outcomes. The application of alternative machine learning algorithms for survival prediction in people living with HIV and AIDS should be explored in future research. Despite the promising utility of RSF in medical studies, few studies have reported its application in Chinese people living with HIV and AIDS. Recently, a study [18] reported the use of a random forest model to predict 10-year survival rates among people living with HIV and AIDS. However, the AUC values of the model at various time points were lower than those of our models. The study relied on secondary data from previously published studies, which limited the selection of candidate predictors and study population, and lacked external validation, thereby limiting the generalization of its findings. Furthermore, it did not translate the prognostic model into clinically applicable tools, such as nomograms, further limiting its practical use. To our knowledge, our study is the first to develop an externally validated RSF model for survival prognosis in Chinese people living with HIV and AIDS.

Some variables previously considered important predictors of survival in people living with HIV and AIDS were not included in our model based on the VIMP criterion. For instance, previous studies have reported poorer treatment outcomes for female patients compared to male patients due to physiological and sociocultural differences. However, in this study, sex was found to be the least important variable based on the VIMP criterion. This may be attributed to the significantly lower number of female individuals living with HIV and AIDS in China and insufficient representation of female patients in this study, limiting the manifestation of this variable’s importance. Viral load, an established risk factor for increased mortality in individuals infected with HIV [4,32], is associated with faster immune system damage and an elevated risk of opportunistic infections. In our study, viral load was excluded from the main analysis due to a high proportion of missing data. However, sensitivity analyses indicated that the exclusion of viral load from the final model did not compromise the model’s performance (Multimedia Appendix 9).

In this study, we comprehensively compared the discriminative ability, calibration, and clinical benefit differences between RSF and Cox models in both the internal and external validation sets. In the internal validation set, the RSF model demonstrated a higher C index, iAUC values, and tdAUC values at all specific time points. It also showed a higher consistency between the predicted results and the observed survival rates, lower iBS values, and exhibited better clinical benefits. These results indicated that the RSF model possessed superior discriminative ability, calibration, and clinical benefits compared to the Cox model. In the external validation set, although all performance metrics of the RSF model declined compared to the internal validation set, several metrics remained slightly superior to those of the Cox model, suggesting that the RSF model demonstrated slightly superior predictive performance, particularly in long-term prognosis assessment. Moreover, we applied a stepwise selection and incorporated restricted cubic splines to variables included in the Cox model. The Cox model’s performance remained inferior to that of the RSF model (Multimedia Appendices 10 and 11).

### Enhance Clinical Utility

Clinical application is a crucial step in translating predictive models from theoretical constructs and formulas into clinical practice, requiring thorough consideration of database representativeness, predictor accessibility, and model practicality. Regrading database representativeness, this study leveraged data from the HIV and AIDS epidemic surveillance system of NCAIDS/STD to develop the predictive models. This database covers information on demographics, treatment, and outcomes of people living with HIV and AIDS from all cities across China. The variables collected in this database are uniformly defined and consistently measured, and all municipal CDCs can access information within their jurisdictions, ensuring broad coverage and standardization across regions. Regarding the predictor accessibility, the 7 predictors included in this model are routinely monitored and reported in the daily work of CDCs. These predictors are objective indicators with established and cost-effective detection methods. Some indicators with higher costs for detection, such as viral load, were not included in the model. Similarly, variables such as failure of first-line antiretroviral therapy (ART) were excluded from the model because we aimed to predict the survival outcomes of people living with HIV and AIDS after the initiation of HAART. In addition, to address the limitation of machine learning algorithms being difficult to be applied in clinical settings, we developed a web-based, interactive prediction model. This interface offers personalized predictions for survival in people living with HIV and AIDS, allowing researchers and clinicians to apply the model in their routine practice.

Given the favorable predictive performance of the RSF model developed in this study, its potential for informing clinical decision-making warrants further exploration through model optimization. Such efforts could advance the precision management of people living with HIV and AIDS by providing both theoretical support and practical tools. First, for individuals at a high risk of mortality, differentiated management strategies could be implemented, such as prioritizing drug resistance testing, initiating ART at an earlier stage [33,34], and integrating psychological interventions with community-based support services [35], to enhance treatment adherence and delay disease progression. Second, the RSF model could be integrated into hospital information systems to enable real-time individual risk assessment and automated alerts, thereby assisting clinicians in timely adjustments of treatment strategies. For example, earlier initiation of second-line ART [36] or adoption of optimized regimens based on dolutegravir could be considered [37], ultimately improving the scientific basis and timeliness of clinical interventions. In addition, in light of the uneven distribution of HIV prevention and treatment resources in China, this model could also inform the precise allocation of medical resources. For instance, patients with a predicted low 5-year survival probability could be prioritized for access to newer, more potent integrase inhibitors and targeted for intensified screening of opportunistic infections (eg, tuberculosis and cryptococcal meningitis) [38,39], thereby maximizing resource efficiency. Finally, the model’s superior predictive performance in older people living with HIV and AIDS underscores its potential utility in guiding targeted interventions for this population. For older individuals at a high risk of mortality, comprehensive application of the aforementioned strategies may help reduce HIV-related mortality and contribute to the overall goal of improving outcomes in vulnerable populations affected by the HIV epidemic in China in recent years [40,41].

### Strengths and Limitations

In this study, we rigorously assessed and minimized the risk of bias in accordance with the Prediction model Risk of Bias Assessment Tool [42]. The study population was sourced from the HIV and AIDS epidemic surveillance system of the NCAIDS/STD, using registry cohort data that adhered to clear and uniform inclusion and exclusion criteria, ensuring the representativeness of people living with HIV and AIDS nationwide. Our predictive model accurately reflected the relationship between predictors and all-cause mortality outcomes. All predictors were consistently defined and measured using standardized methods, thereby ensuring the reliability and accessibility of the predictors. The outcomes for all study participants were uniformly determined using the guideline-recommended method, and all participants had at least one follow-up record, ensuring the validity of the outcomes. In data analysis, we ensured adequate sample sizes for both model development and external validation and avoided categorizing continuous variables to present overestimating model performance. In addition, this study used a resampling method combining oversampling and undersampling to address class imbalance, which reduced selection bias, improved statistical efficiency, and ensured the robustness and reliability of the model [43]. We comprehensively assessed the model’s discrimination, calibration, and clinical utility. Meanwhile, we conducted multiple sensitivity analyses to avoid overestimating model performance. Despite statistical differences in nearly all candidate variables between the internal and external validation cohorts, the model demonstrated strong validation performance, confirming its generalizability.

The limitations of this study are as follows. First, the relatively low positive predictive value (Multimedia Appendix 7) of the RSF model suggests that a substantial proportion of individuals classified as high risk may actually have a low risk of mortality, thereby limiting the model’s ability in accurately identifying high-risk patients. Consequently, the RSF model should not be used as the sole basis for initiating intensive interventions, such as adjustments to ART. In clinical applications, intervention decisions should be informed by a combination of this model, other validated risk assessment tools, and individualized clinical judgment. Future studies should focus on improving the model’s positive predictive value by optimizing its algorithmic framework and integrating additional clinical variables and multisource data, thereby enhancing its utility and reliability in high-risk patient identification and clinical decision support. Second, we used data exclusively from regional databases of the NCAIDS/STD, and only 7 variables were included in the model development. Some potential factors that may be associated with the prognosis of people living with HIV and AIDS were not included in this research. Third, we did not include people living with HIV and AIDS aged <18 years. Due to differences between adult and pediatric patients, our findings may not be generalizable to pediatric patients.

### Conclusions

In this study, a machine learning–based RSF model offered personalized and accurate survival prediction and remarkable prognostic stratification for people living with HIV and AIDS following the initiation of HAART in China. The RSF algorithm may outperform the traditional Cox model in survival prediction for people living with HIV and AIDS. The survival prediction tool constructed in this study may improve clinical decision-making for people living with HIV and AIDS.

## Appendix

Multimedia Appendix 1 TRIPOD (Transparent Reporting of a Multivariable Prediction Model for Individual Prognosis or Diagnosis) statement checklist.

Multimedia Appendix 2 The flowchart of participant selection.

Multimedia Appendix 3 Definitions and coding of variables.

Multimedia Appendix 4 The number and proportion of missing data of each variable.

Multimedia Appendix 5 Comparison of area under the curve performance between random survival forest and Cox proportional hazards models, evaluated by DeLong tests in the internal and external validation sets.

Multimedia Appendix 6 Predicted survival rates in the random survival forest models by risk groups in the internal and external validation sets.

Multimedia Appendix 7 Validity and reliability of the random survival forest model and the Cox proportional hazards model at 1, 3, 5, and 8 years following the initiation of highly active antiretroviral therapy in the internal and external validation sets.

Multimedia Appendix 8 Performance comparison of random survival forest model versus Cox proportional hazards model, evaluated by Fisher exact test for sensitivity in the internal and external validation sets.

Multimedia Appendix 9 Sensitivity analysis 1: net reclassification improvement comparing the random survival forest (RSF) models that included log-transformed viral load and the RSF models that included non–log-transformed viral load in the internal and external validation sets.

Multimedia Appendix 10 Sensitivity analysis 2: Cox proportional hazards models using the stepwise method.

Multimedia Appendix 11 Sensitivity analysis 3: comparative analysis of random survival forest models and Cox proportional hazards models for predicting outcomes, with emphasis on linear variable transformation and selection.

Multimedia Appendix 12 Sensitivity analysis 4: the performance of the random survival forest models by age groups in the internal and external validation sets.

## Abbreviations

ART: antiretroviral therapy
AUC: area under the curve
BG: blood glucose
C index: consistency index
CDC: Centers for Disease Control and Prevention
CD4: CD4 T lymphocyte count
DCA: decision curve analysis
HAART: highly active antiretroviral therapy
iAUC: integrated area under the curve
iBS: integrated Brier score
NCAIDS/STD: National Center for AIDS/STD Control and Prevention
NRI: net reclassification improvement
ROC: receiver operating characteristic
RSF: random survival forest
tdAUC: time-dependent area under the curve
VIMP: variable importance
WBC: white blood cell
WHO: World Health Organization

## Data Availability

Study data will not be publicly available. Data can be made available by the corresponding author to any interested researcher following approval of a concept sheet summarizing the analysis to be done. Deidentified participant data and a data dictionary can be made available and shared under a data transfer agreement. Requests for access to the study data should be sent to the corresponding author by email.
