# Supplementary figures and images for: Development and Spatial External Validation of a Predictive Model of Survival Based on Random Survival Forest Analysis for People Living With HIV and AIDS After Highly Active Antiretroviral Therapy in China: Retrospective Cohort Study
Source: J Med Internet Res. 2025 Jun 2;27:e71257. doi: 10.2196/71257 (PMC12171649; doi:10.2196/71257)

**Multimedia Appendix 2. The flow chart of participant selection**


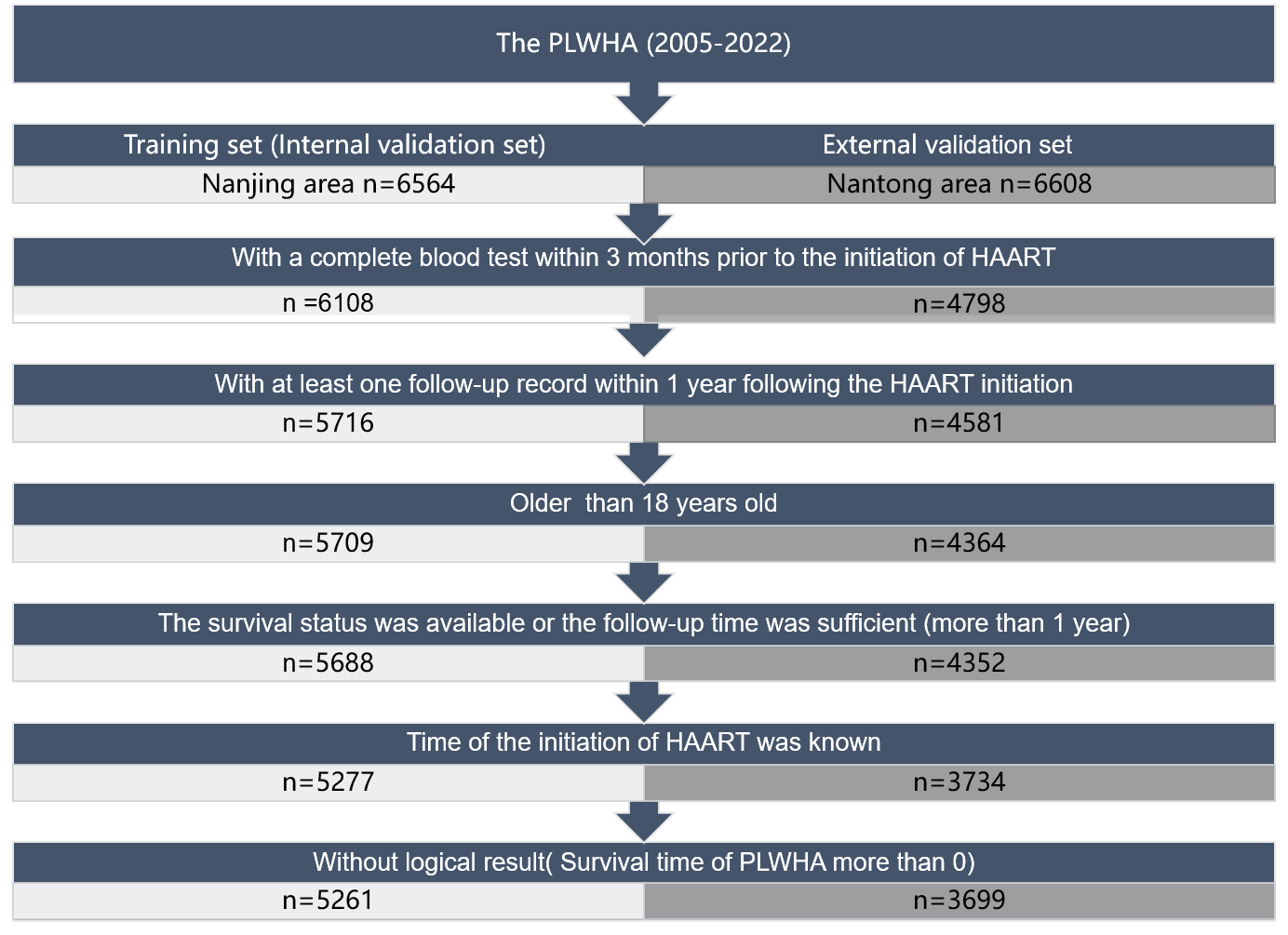

Supplement: Multimedia Appendix 2 [file jmir_v27i1e71257_app2.docx]
