# Supplementary material for: Development and Spatial External Validation of a Predictive Model of Survival Based on Random Survival Forest Analysis for People Living With HIV and AIDS After Highly Active Antiretroviral Therapy in China: Retrospective Cohort Study
Source: J Med Internet Res. 2025 Jun 2;27:e71257. doi: 10.2196/71257 (PMC12171649; doi:10.2196/71257)
Supplement: Multimedia Appendix 3 [file jmir_v27i1e71257_app3.docx]

**Multimedia Appendix 3.** **Definitions and coding of variables**

| **Variable** | **Definition** | **Coding** |
| --- | --- | --- |
| **Demographic characteristic** | | |
| Age (years) | Age at the initiation of HAART | Continuous variable |
| Sex | Sex | 1=Male  2=Female |
| Marital status | Marital status at the initiation of HAART | 1=Unmarried  2=Married |
| Education level | Education level at the initiation of HAART | 1=Illiterate or primary school  2=Middle school  3=High school  4=College or above |
| BMI (kg/m^2^) | Body mass index at the initiation of HAART | 1=＜18.5  2 =18.5-23.9  3=＞23.9 |
| **Clinical characteristic** | | |
| Infection route | Infection route of HIV/AIDS | 1=Homosexual transmission  2=Heterosexual transmission  3=Other |
| History of STD | History of sexually transmitted diseases | 0=No  1=Yes |
| WHO clinical stage | WHO clinical classification of HIV/AIDS at the initiation of HAART | 1=I  2=II  3=III  4=IV |
| **Biochemical index** | | |
| CD4 (cells/μL) | Recent CD4 cell count within 3 months prior to the initiation of HAART | Continuous variable |
| BG (mmol/L) | Recent Blood glucose within 3 months prior to the initiation of HAART | Continuous variable |
| WBC (10^9^/L) | White blood cell count within 3 months prior to the initiation of HAART | Continuous variable |
| PLT (10^9^/L) | Platelet within 3 months prior to the initiation of HAART | Continuous variable |
| HB (g/L) | Hemoglobin within 3 months prior to the initiation of HAART | Continuous variable |
| SCr (μmol/L) | Serum creatinine within 3 months prior to the initiation of HAART | Continuous variable |
| ALT (U/L) | Alanine aminotransferase within 3 months prior to the initiation of HAART | Continuous variable |
| TBIL (μmol/L) | Total bilirubin within 3 months prior to the initiation of HAART | Continuous variable |
| HAART regimen | Medicine type within 3 months prior to the initiation of HAART | 1 =NRTls/PIls/Mix  2=NNRTIs  3=INSTls |
| TG* (mmol/L) | Triglyceride within 3 months prior to the initiation of HAART | Continuous variable |
| TC* (mmol/L) | Serum total cholesterol within 3 months prior to the initiation of HAART | Continuous variable |
| Viral load (copies/mL) | Recent Viral load within 3 months prior to the initiation of HAART | Continuous variable |

Abbreviation: ALT: Alanine aminotransferase; BG: Blood glucose; BMI: Body mass index; HAART: Highly active anti-retroviral therapy; HB: Haemoglobin; INSTls: Integraseinhibitors; Mix: Different types of medicine compound preparation; NNRTIs: Non-nucleoside reverse transcriptase inhibitor; NRTIs: Nucleotide reverse transcriptase inhibitor; Pils: Protease inhibitor; PLT: Platelet; SCr: Serum creatinine; TBIL: Total bilirubin; TC: Total cholesterol; TG: Triglyceride; WBC: [White blood cell](https://www.baidu.com/s?wd=white%20blood%20cell&rsv_idx=2&tn=baiduhome_pg&usm=4&ie=utf-8&rsv_pq=9276ef01001d7dc1&oq=WBC%E5%85%A8%E7%A7%B0&rsv_t=981ebkmlxgw0r%2BTcuVwH6Cn2WekSkylZuBgxfjdy%2BADny2pq3NzhEjnK%2B1ogSi%2Fz2zfA&sa=re_dqa_zy&icon=1); WHO: World Health Organization.

TC* and TG* were not included as candidates in the model due to missing data exceeding 20%.
