# Supplementary material for: Development and Spatial External Validation of a Predictive Model of Survival Based on Random Survival Forest Analysis for People Living With HIV and AIDS After Highly Active Antiretroviral Therapy in China: Retrospective Cohort Study
Source: J Med Internet Res. 2025 Jun 2;27:e71257. doi: 10.2196/71257 (PMC12171649; doi:10.2196/71257)
Supplement: Multimedia Appendix 4 [file jmir_v27i1e71257_app4.docx]

**Multimedia Appendix 4. The number and proportion of missing data of each variable**

| **Variable** | **Training set**  **(*n*=5261)** | **External validation set**  **(*n*=3699)** |
| --- | --- | --- |
| **Demographic characteristic** | | |
| Age (year) | 0 (0.0) | 0 (0.0) |
| Sex | 0 (0.0) | 0 (0.0) |
| Male |  |  |
| Female |  |  |
| Marital status | 26 (0.5) | 9 (0.2) |
| Unmarried |  |  |
| Married |  |  |
| Education | 0 (0.0) | 0 (0.0) |
| Illiterate or primary school |  |  |
| Middle school |  |  |
| High school |  |  |
| College and above |  |  |
| BMI | 0 (0.0) | 0 (0.0) |
| < 18.5 |  |  |
| 18.5-23.9 |  |  |
| ＞23.9 |  |  |
| **Clinical characteristic** | | |
| Infection route | 19 (0.4) | 13 (0.4) |
| Homosexual transmission |  |  |
| Heterosexual transmission |  |  |
| Other transmission |  |  |
| History of STD | 1008 (19.2) | 195 (5.3) |
| No |  |  |
| Yes |  |  |
| WHO clinical stage | 2 (0.0) | 0 (0.0) |
| Ⅰ |  |  |
| Ⅱ |  |  |
| Ⅲ |  |  |
| Ⅳ |  |  |
| HAART regimen | 0 (0.0) | 0 (0.0) |
| NRTIs or PiIs or Mix |  |  |
| NNRTIs |  |  |
| INSTls |  |  |
| **Biochemical index** | | |
| CD4 (cells/μL) | 767 (14.5) | 11 (0.3) |
| BG (mmol/L) | 666 (12.6) | 185 (5.0) |
| WBC (10^9^/L) | 227 (4.3) | 109 (2.9) |
| PLT (10^9^/L) | 230 (4.3) | 121 (3.3) |
| HB (g/L) | 235 (4.4) | 120 (3.2) |
| SCr (μmol/L) | 591 (11.2) | 306 (8.3) |
| ALT (U/L) | 220 (4.1) | 115 (3.1) |
| TBIL (umol/L) | 288 (5.4) | 161 (4.4) |
| TG* (mmol/L) | 1090 (20.7) | 1677 (45.3) |
| TC* (mmol/L) | 1098 (20.8) | 1688 (45.6) |
| Viral load* (copies/mL) | 2566 (48.7) | 1403 (37.9) |

Abbreviation: ALT: Alanine aminotransferase; BG: Blood glucose; BMI: Body mass index; HAART: Highly active anti-retroviral therapy; HB: Haemoglobin; INSTls: Integraseinhibitors; Mix: Different types of medicine compound preparation; NNRTIs: Non-nucleoside reverse transcriptase inhibitor; NRTIs: Nucleotide reverse transcriptase inhibitor; Pils: Protease inhibitor; PLT: Platelet; SCr: Serum creatinine; TBIL: Total bilirubin; TC: Total cholesterol; TG: Triglyceride; WBC: [White blood cell](https://www.baidu.com/s?wd=white%20blood%20cell&rsv_idx=2&tn=baiduhome_pg&usm=4&ie=utf-8&rsv_pq=9276ef01001d7dc1&oq=WBC%E5%85%A8%E7%A7%B0&rsv_t=981ebkmlxgw0r%2BTcuVwH6Cn2WekSkylZuBgxfjdy%2BADny2pq3NzhEjnK%2B1ogSi%2Fz2zfA&sa=re_dqa_zy&icon=1); WHO: World Health Organization.

TC*, TG* and Viral load* were not included as candidates in the model due to missing data exceeding 20%.
