# Supplementary material for: Development and Spatial External Validation of a Predictive Model of Survival Based on Random Survival Forest Analysis for People Living With HIV and AIDS After Highly Active Antiretroviral Therapy in China: Retrospective Cohort Study
Source: J Med Internet Res. 2025 Jun 2;27:e71257. doi: 10.2196/71257 (PMC12171649; doi:10.2196/71257)
Supplement: Multimedia Appendix 5 [file jmir_v27i1e71257_app5.docx]

**Multimedia Appendix 6.** **Comparison of AUC performance between RSF and Cox Models: evaluated by DeLong tests in the internal and external validation sets**

| **Data set** | **model** | **RSF model**  **(1-year)** | **RSF model**  **(3-year)** | **RSF model**  **(5-year)** | **RSF model**  **(8-year)** |
| --- | --- | --- | --- | --- | --- |
| Internal validation set | Cox model (1-year) | *P*<0.001 | - | - | - |
|  | Cox model (3-year) | - | *P*<0.001 |  | - |
|  | Cox model (5-year) | - | - | *P*<0.001 | - |
|  | Cox model (8-year) | - | - | - | *P*<0.001 |
| External validation set | Cox model (1-year) | *P*=0.050 | - | - | - |
|  | Cox model (3-year) | - | *P*=0.053 |  | - |
|  | Cox model (5-year) | - | - | *P*=0.003 | - |
|  | Cox model (8-year) | - | - | - | *P*=0.102 |

Abbreviations: RSF: random survival forest; Cox: Cox proportional hazards
