# Supplementary material for: Development and Spatial External Validation of a Predictive Model of Survival Based on Random Survival Forest Analysis for People Living With HIV and AIDS After Highly Active Antiretroviral Therapy in China: Retrospective Cohort Study
Source: J Med Internet Res. 2025 Jun 2;27:e71257. doi: 10.2196/71257 (PMC12171649; doi:10.2196/71257)
Supplement: Multimedia Appendix 6 [file jmir_v27i1e71257_app6.docx]

**Multimedia Appendix 5. Predicted survival rates in the RSF models by risk groups in the internal and external validation sets**

| **Group** | **Time** | **Internal validation data** |  | **External validation data** |
| --- | --- | --- | --- | --- |
|  |  | **Survival rate (95%CI)** |  | **Survival rate (95%CI)** |
| Low risk | 1-year | 0.987 (0.980 - 0.993) |  | 0.993 (0.989 - 0.997) |
|  | 3-year | 0.972 (0.962 - 0.981) |  | 0.989 (0.984 - 0.995) |
|  | 5-year | 0.946 (0.932 - 0.960) |  | 0.982 (0.974 - 0.989) |
|  | 8-year | 0.916 (0.896 - 0.938) |  | 0.972 (0.960 - 0.984) |
| Medium risk | 1-year | 0.827 (0.793 - 0.863) |  | 0.956 (0.946 - 0.966) |
|  | 3-year | 0.544 (0.498 - 0.595) |  | 0.926 (0.913 - 0.940) |
|  | 5-year | 0.451 (0.403 - 0.506) |  | 0.894 (0.876 - 0.912) |
|  | 8-year | 0.388 (0.337 - 0.447) |  | 0.867 (0.846 - 0.889) |
| High risk | 1-year | 0.324 (0.266 - 0.395) |  | 0.884 (0.853 - 0.918) |
|  | 3-year | 0.144 (0.102 - 0.202) |  | 0.813 (0.773 - 0.855) |
|  | 5-year | 0.128 (0.088 - 0.186) |  | 0.796 (0.752 - 0.842) |
|  | 8-year | 0.083 (0.048 - 0.143) |  | 0.768 (0.717 - 0.823) |
| Low risk | 1-year | 0.963 (0.953 - 0.972) |  | 0.980 (0.975 - 0.985) |
|  | 3-year | 0.929 (0.916 - 0.943) |  | 0.967 (0.960 - 0.973) |
|  | 5-year | 0.884 (0.866 - 0.903) |  | 0.948 (0.939 - 0.958) |
|  | 8-year | 0.851 (0.828 - 0.874) |  | 0.931 (0.919 - 0.944) |
| High risk | 1-year | 0.544 (0.497 - 0.596) |  | 0.902 (0.879 - 0.924) |
|  | 3-year | 0.209 (0.171 - 0.255) |  | 0.840 (0.812 - 0.870) |
|  | 5-year | 0.181 (0.144 - 0.227) |  | 0.814 (0.781 - 0.848) |
|  | 8-year | 0.121 (0.087 - 0.167) |  | 0.789 (0.750 - 0.829) |

Abbreviation: RSF: random survival forest
