# Supplementary material for: Development and Spatial External Validation of a Predictive Model of Survival Based on Random Survival Forest Analysis for People Living With HIV and AIDS After Highly Active Antiretroviral Therapy in China: Retrospective Cohort Study
Source: J Med Internet Res. 2025 Jun 2;27:e71257. doi: 10.2196/71257 (PMC12171649; doi:10.2196/71257)
Supplement: Multimedia Appendix 7 [file jmir_v27i1e71257_app7.docx]

**Multimedia Appendix 7.** **Validity and reliability of the RSF model and the Cox model at 1, 3, 5, 8 years following the initiation of HAART in the internal and external validation sets**

| **Data set** | **Performance index** | **RSF model** | | | |  | **Cox model** | | | |
| --- | --- | --- | --- | --- | --- | --- | --- | --- | --- | --- |
|  |  | **1 - year** | **3 - year** | **5 - year** | **8 - year** |  | **1 - year** | **3 - year** | **5 - year** | **8 - year** |
| Internal validation  set | Sensitivity  (True Positive Rate) | 0.983  (0.977 - 0.989) | 0.990  (0.986 - 0.994) | 0.982  (0.976 - 0.988) | 0.975  (0.968 - 0.982) |  | 0.940  (0.930 - 0.951) | 0.950  (0.940 - 0.959) | 0.947  (0.937 - 0.956) | 0.942  (0.932 - 0.952) |
|  | Specificity  (True Negative Rate) | 0.565  (0.543 - 0.587) | 0.623  (0.602 - 0.644) | 0.640  (0.619 - 0.661) | 0.651  (0.630 - 0.672) |  | 0.559  (0.537 - 0.580) | 0.612  (0.591 - 0.634) | 0.629  (0.608 - 0.650) | 0.640  (0.619 - 0.661) |
|  | False Negative Rate | 0.017  (0.011 - 0.023) | 0.010  (0.006 - 0.014) | 0.018  (0.012 - 0.024) | 0.025  (0.018 - 0.032) |  | 0.060  (0.049 - 0.070) | 0.050  (0.041 - 0.060) | 0.054  (0.044 - 0.063) | 0.058  (0.048 - 0.069) |
|  | False Positive Rate | 0.435  (0.413 - 0.457) | 0.377  (0.356 - 0.399) | 0.360  (0.339 - 0.381) | 0.349  (0.328 - 0.370) |  | 0.441  (0.420 - 0.463) | 0.388  (0.367 - 0.409) | 0.371  (0.350 - 0.392) | 0.360  (0.339 - 0.381) |
|  | Youden’s Index | 0.548  (0.526 - 0.570) | 0.613  (0.591 - 0.634) | 0.622  (0.601 - 0.644) | 0.626  (0.605 - 0.647) |  | 0.499  (0.477 - 0.521) | 0.562  (0.540 - 0.584) | 0.576  (0.554 - 0.598) | 0.582  (0.560 - 0.603) |
|  | Positive  Predictive Value | 0.231  (0.213 - 0.250) | 0.395  (0.374 - 0.417) | 0.441  (0.420 - 0.463) | 0.470  (0.448 - 0.491) |  | 0.221  (0.203 - 0.239) | 0.379  (0.358 - 0.400) | 0.425  (0.403 - 0.447) | 0.453  (0.431 - 0.475) |
|  | Negative  Predictive Value | 0.996  (0.993 - 0.999) | 0.996  (0.993 - 0.999) | 0.992  (0.988 - 0.996) | 0.988  (0.983 - 0.993) |  | 0.986  (0.981 - 0.991) | 0.980  (0.974 - 0.986) | 0.976  (0.969 - 0.983) | 0.972  (0.965 - 0.979) |
|  | Positive  Likelihood Ratio | 2.259  (2.134 - 2.384) | 2.624  (2.437 - 2.811) | 2.730  (2.523 - 2.937) | 2.795  (2.575 - 3.014) |  | 2.131  (2.025 - 2.236) | 2.449  (2.293 - 2.604) | 2.553  (2.379 - 2.727) | 2.615  (2.430 - 2.801) |
|  | Negative  Likelihood Ratio | 0.030  (0.023 - 0.038) | 0.016  (0.011 - 0.022) | 0.028  (0.021 - 0.035) | 0.038  (0.030 - 0.047) |  | 0.107  (0.093 - 0.120) | 0.082  (0.070 - 0.094) | 0.085  (0.073 - 0.097) | 0.091  (0.078 - 0.104) |
|  | Kappa Value | 0.721  (0.701 - 0.741) | 0.766  (0.748 - 0.785) | 0.778  (0.760 - 0.796) | 0.785  (0.767 - 0.803) |  | 0.713  (0.693 - 0.733) | 0.754  (0.735 - 0.772) | 0.765  (0.747 - 0.784) | 0.772  (0.753 - 0.790) |
|  | Agreement Rate | 0.614  (0.593 - 0.635) | 0.696  (0.676 - 0.716) | 0.717  (0.697 - 0.737) | 0.729  (0.710 - 0.749) |  | 0.604  (0.582 - 0.625) | 0.680  (0.659 - 0.700) | 0.701  (0.680 - 0.721) | 0.713  (0.693 - 0.732) |
| External validation  set | Sensitivity  (True Positive Rate) | 0.862  (0.851 - 0.873) | 0.875  (0.864 - 0.886) | 0.849  (0.837 - 0.860) | 0.842  (0.830 - 0.854) |  | 0.805  (0.792 - 0.818) | 0.828  (0.816 - 0.840) | 0.792  (0.779 - 0.805) | 0.787  (0.773 - 0.800) |
|  | Specificity  (True Negative Rate) | 0.513  (0.497 - 0.529) | 0.521  (0.505 - 0.537) | 0.523  (0.507 - 0.540) | 0.525  (0.509 - 0.541) |  | 0.511  (0.495 - 0.527) | 0.518  (0.502 - 0.534) | 0.520  (0.504 - 0.536) | 0.521  (0.505 - 0.537) |
|  | False Negative Rate | 0.138  (0.127 - 0.149) | 0.125  (0.114 - 0.136) | 0.152  (0.140 - 0.163) | 0.158  (0.146 - 0.170) |  | 0.195  (0.182 - 0.208) | 0.172  (0.160 - 0.184) | 0.208  (0.195 - 0.221) | 0.213  (0.200 - 0.227) |
|  | False Positive Rate | 0.487  (0.471 - 0.504) | 0.479  (0.463 - 0.495) | 0.477  (0.461 - 0.493) | 0.475  (0.459 - 0.491) |  | 0.489  (0.473 - 0.506) | 0.482  (0.466 - 0.498) | 0.480  (0.464 - 0.497) | 0.479  (0.463 - 0.495) |
|  | Youden’s Index | 0.374  (0.359 - 0.390) | 0.396  (0.380 - 0.411) | 0.372  (0.356 - 0.387) | 0.367  (0.352 - 0.383) |  | 0.316  (0.301 - 0.331) | 0.346  (0.331 - 0.362) | 0.312  (0.297 - 0.327) | 0.308  (0.293 - 0.323) |
|  | Positive  Predictive Value | 0.057  (0.050 - 0.065) | 0.091  (0.082 - 0.100) | 0.106  (0.096 - 0.116) | 0.115  (0.105 - 0.126) |  | 0.054  (0.046 - 0.061) | 0.086  (0.077 - 0.095) | 0.099  (0.089 - 0.109) | 0.108  (0.098 - 0.118) |
|  | Negative  Predictive Value | 0.991  (0.988 - 0.994) | 0.987  (0.983 - 0.991) | 0.981  (0.977 - 0.986) | 0.978  (0.974 - 0.983) |  | 0.987  (0.983 - 0.991) | 0.982  (0.978 - 0.986) | 0.974  (0.969 - 0.979) | 0.971  (0.965 - 0.976) |
|  | Positive  Likelihood Ratio | 1.768  (1.724 - 1.812) | 1.826  (1.777 - 1.874) | 1.780  (1.735 - 1.825) | 1.773  (1.729 - 1.817) |  | 1.645  (1.611 - 1.679) | 1.719  (1.679 - 1.758) | 1.649  (1.615 - 1.684) | 1.643  (1.609 - 1.677) |
|  | Negative  Likelihood Ratio | 0.270  (0.255 - 0.284) | 0.240  (0.226 - 0.254) | 0.290  (0.275 - 0.304) | 0.301  (0.286 - 0.316) |  | 0.382  (0.367 - 0.398) | 0.332  (0.317 - 0.347) | 0.400  (0.384 - 0.416) | 0.410  (0.394 - 0.425) |
|  | Kappa Value | 0.676  (0.661 - 0.691) | 0.682  (0.667 - 0.697) | 0.683  (0.668 - 0.698) | 0.684  (0.669 - 0.699) |  | 0.673  (0.658 - 0.688) | 0.678  (0.663 - 0.693) | 0.678  (0.663 - 0.693) | 0.678  (0.663 - 0.693) |
|  | Agreement Rate | 0.524  (0.508 - 0.540) | 0.539  (0.523 - 0.555) | 0.544  (0.528 - 0.559) | 0.547  (0.531 - 0.563) |  | 0.520  (0.504 - 0.537) | 0.534  (0.518 - 0.550) | 0.537  (0.521 - 0.553) | 0.539  (0.523 - 0.555) |

Abbreviations: RSF: random survival forest; Cox: Cox proportional hazards
