# Supplementary material for: Development and Spatial External Validation of a Predictive Model of Survival Based on Random Survival Forest Analysis for People Living With HIV and AIDS After Highly Active Antiretroviral Therapy in China: Retrospective Cohort Study
Source: J Med Internet Res. 2025 Jun 2;27:e71257. doi: 10.2196/71257 (PMC12171649; doi:10.2196/71257)
Supplement: Multimedia Appendix 8 [file jmir_v27i1e71257_app8.docx]

**Multimedia Appendix 8. Performance comparison of RSF model versus Cox model, evaluated by Fisher's exact test for sensitivity in the internal and external validation sets**

| **Data set** | **model** | **RSF model (1-year)** | |  | **RSF model (3-year)** | |  | **RSF model (5-year)** | |  | **RSF model (8-year)** | |
| --- | --- | --- | --- | --- | --- | --- | --- | --- | --- | --- | --- | --- |
|  |  | **Statistics** | ***P* value** |  | **Statistics** | ***P* value** |  | **Statistics** | ***P* value** |  | **Statistics** | ***P* value** |
| Internal validation  set | Cox model (1-year) | 4.68 | <0.05 |  |  |  |  | - | - |  | - | - |
|  | Cox model (3-year) | - | - |  | 9.67 | <0.001 |  | - | - |  | - | - |
|  | Cox model (5-year) | - | - |  | - | - |  | 7.29 | <0.05 |  | - | - |
|  | Cox model (8-year) | - | - |  | - | - |  | - | - |  | 5.87 | <0.05 |
| External validation  set | Cox model (1-year) | 1.05 | 0.30 |  |  |  |  | - | - |  | - | - |
|  | Cox model (3-year) | - | - |  | 1.32 | 0.25 |  | - | - |  | - | - |
|  | Cox model (5-year) | - | - |  | - | - |  | 2.11 | 0.146 |  | - | - |
|  | Cox model (8-year) | - | - |  | - | - |  | - | - |  | 2.21 | 0.137 |

Abbreviations: Cox: Cox proportional hazards; RSF: random survival forest.

**Performance comparison of RSF model versus Cox model, evaluated by Fisher's exact test for specificity in the internal and external validation sets**

| **Data set** | **model** | **RSF model (1-year)** | |  | **RSF model (3-year)** | |  | **RSF model (5-year)** | |  | **RSF model (8-year)** | |
| --- | --- | --- | --- | --- | --- | --- | --- | --- | --- | --- | --- | --- |
|  |  | **Statistics** | ***P* value** |  | **Statistics** | ***P* value** |  | **Statistics** | ***P* value** |  | **Statistics** | ***P* value** |
| Internal validation  set | Cox model (1-year) | 0.12 | 0.73 |  |  |  |  | - | - |  | - | - |
|  | Cox model (3-year) | - | - |  | 0.34 | 0.56 |  | - | - |  | - | - |
|  | Cox model (5-year) | - | - |  | - | - |  | 0.36 | 0.55 |  | - | - |
|  | Cox model (8-year) | - | - |  | - | - |  | - | - |  | 0.37 | 0.54 |
| External validation  set | Cox model (1-year) | 0.02 | 0.89 |  |  |  |  | - | - |  | - | - |
|  | Cox model (3-year) | - | - |  | 0.04 | 0.85 |  | - | - |  | - | - |
|  | Cox model (5-year) | - | - |  | - | - |  | 0.08 | 0.77 |  | - | - |
|  | Cox model (8-year) | - | - |  | - | - |  | - | - |  | 0.10 | 0.75 |

Abbreviations: Cox: Cox proportional hazards; RSF: random survival forest.
