# Supplementary material for: Development and Spatial External Validation of a Predictive Model of Survival Based on Random Survival Forest Analysis for People Living With HIV and AIDS After Highly Active Antiretroviral Therapy in China: Retrospective Cohort Study
Source: J Med Internet Res. 2025 Jun 2;27:e71257. doi: 10.2196/71257 (PMC12171649; doi:10.2196/71257)
Supplement: Multimedia Appendix 9 [file jmir_v27i1e71257_app9.docx]

**Multimedia Appendix 9. Sensitivity analysis 1: NRIs comparing the RSF models that included log-transformed viral load and the RSF models that included non-log-transformed viral load in the internal and external validation sets**

| **Time** | **Internal validation set** | |  | **External validation set** | |
| --- | --- | --- | --- | --- | --- |
|  | **NRI (log transformed)** | **NRI (non-log transformed)** |  | **NRI (log transformed)** | **NRI (non-log transformed)** |
| 1-year | 0.085 (-0.009 - 0.136) | 0.048 (-0.044 - 0.083) |  | 0.008 (-0.001 - 0.030) | 0.008 (-0.002 - 0.023) |
| 2-year | 0.053 (0.009 - 0.099) | 0.043 (-0.019 - 0.100) |  | -0.006 (-0.032 - 0.034) | -0.001 (-0.024 - 0.018) |
| 3-year | 0.061 (0.009 - 0.094) | 0.055 (-0.009 - 0.099) |  | 0.003 (-0.029 - 0.050) | 0.016 (-0.015 - 0.044) |

Abbreviations: RSF: random survival forest; NRI: net reclassification improvement
