# Supplementary material for: Development and Spatial External Validation of a Predictive Model of Survival Based on Random Survival Forest Analysis for People Living With HIV and AIDS After Highly Active Antiretroviral Therapy in China: Retrospective Cohort Study
Source: J Med Internet Res. 2025 Jun 2;27:e71257. doi: 10.2196/71257 (PMC12171649; doi:10.2196/71257)
Supplement: Multimedia Appendix 11 [file jmir_v27i1e71257_app11.docx]

**Multimedia Appendix 11. Sensitivity analysis 3: Comparative Analysis of RSF Models and Cox Models for Predicting Outcomes, with Emphasis on Linear Variable Transformation and Selection**

Restricted cubic splines were used to fit the non-linear relationship. Three continuous variables which did not satisfy the linear relation, including SCr, BG, and CD4 were transferred into categorical variable. By ranking the importance during the modeling process and the top 6 variables were selected to develop the final RSF model, including hemoglobin volume, age, infection route, education level, white blood cell count, and body mass index.

The Cox model was constructed by the univariate and multivariate analyses. The proportional hazards (PH) assumption was examined using the “survival” package. Variables that met the PH assumption were selected by the stepwise regression method based on Akaike information criterion (AIC). The final Cox model included marital status, history of STD, and CD4.


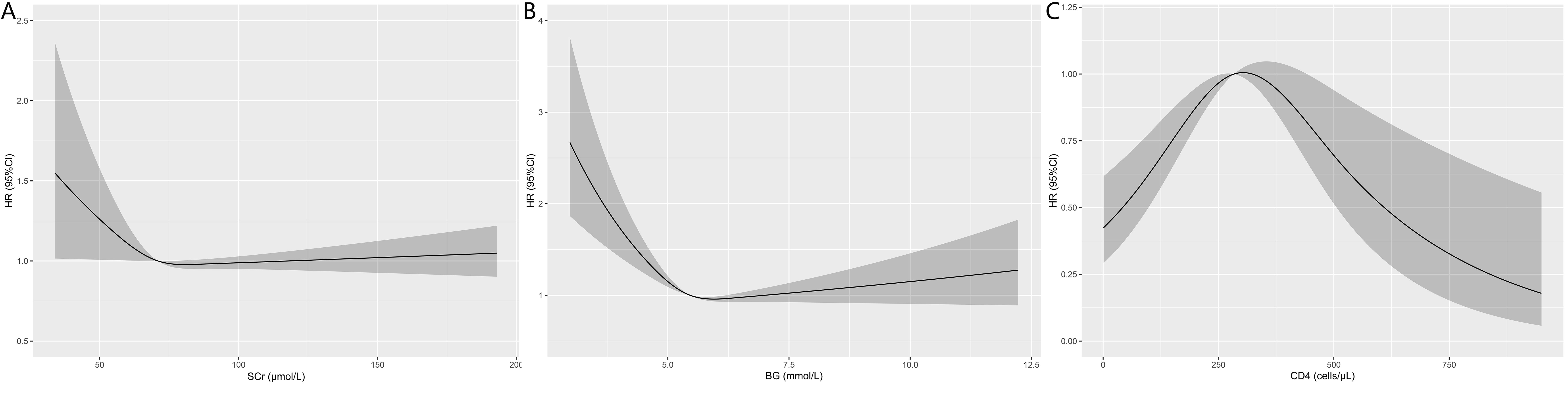


**The restricted cubic splines for the continuous variables indicating a non-linear relationship.** (A) SCr; (B) BG; (C) CD4.

Abbreviations: Blood glucose: CD4: CD4 T lymphocyte count; SCr: Serum Creatinine.


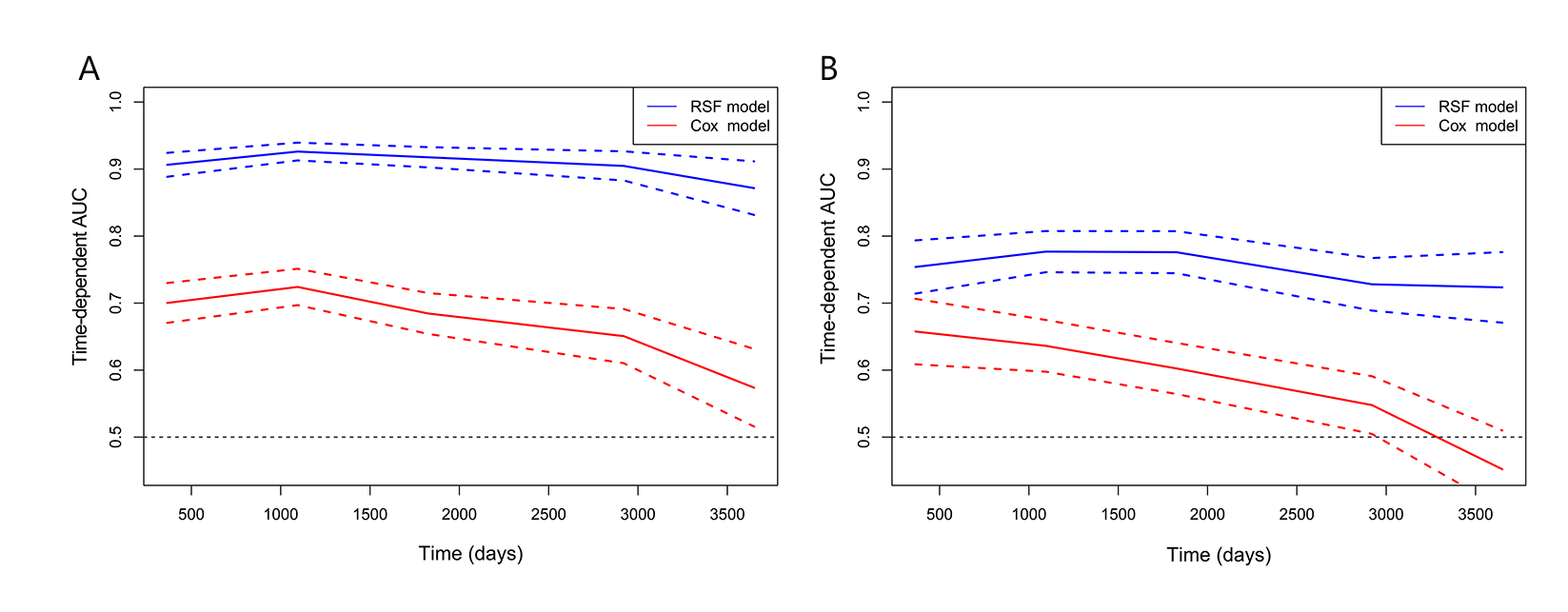


**Comparison of tdAUC curve for RSF and Cox models after linear relationship assessment** in the (A) internal validation set and (B) external validation set.

Abbreviations: Cox: Cox proportional hazards; RSF: random survival forest; tdAUC: time-dependent area under curve.


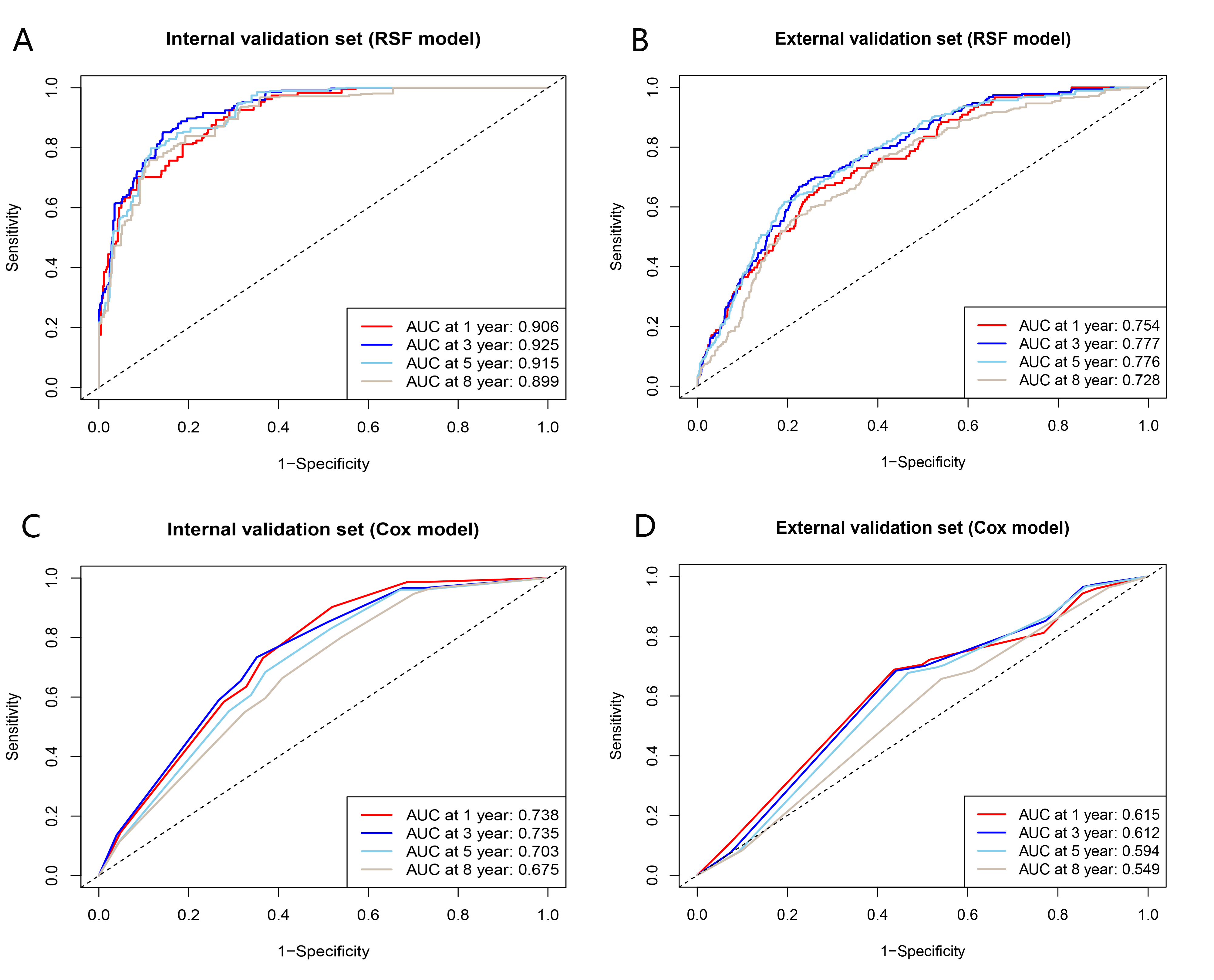


**ROC curves of the RSF models and the Cox models after linear relationship assessment.** (A) RSF model in the internal validation set; (B) RSF model in the external validation set; (C) Cox model in the internal validation set; (D) Cox model in the external validation set.

Abbreviations: Cox: Cox proportional hazards; ROC: receiver operating characteristic; RSF: random survival forest.


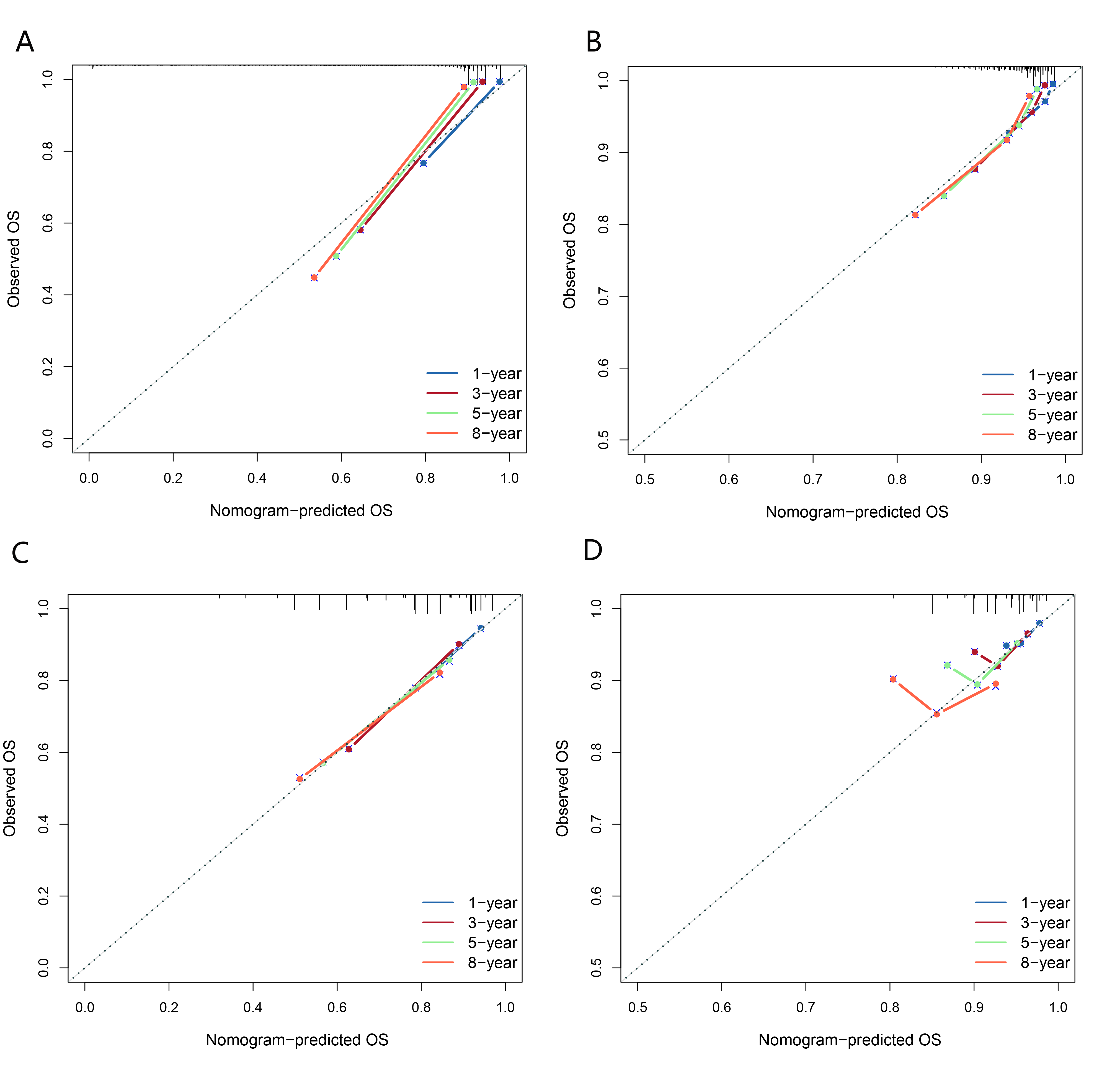


**The calibration curves of RSF model and Cox model after linear relationship assessment.** (A) RSF model in the internal validation set; (B) RSF model in the external validation set; (C) Cox model in the internal validation set; (D) Cox model in the external validation set.

Abbreviations: Cox: Cox proportional hazards; RSF: random survival forest.

**
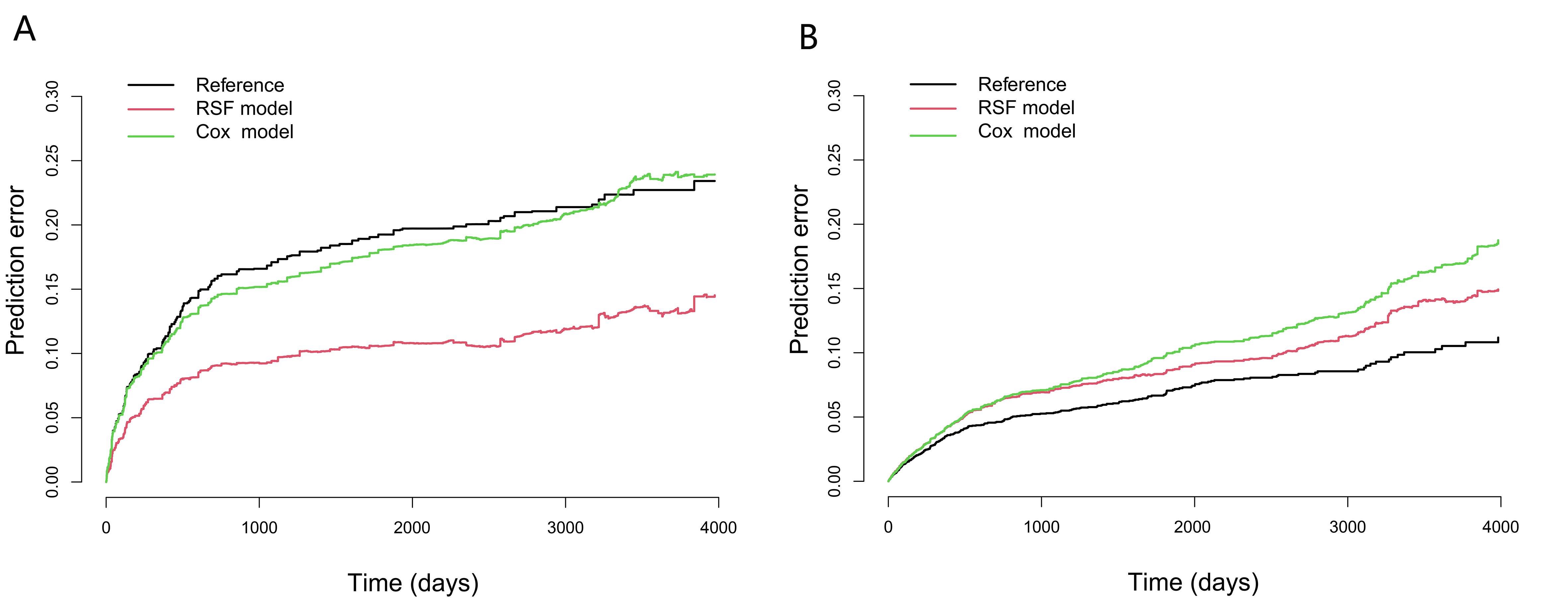
**

**The brier score curves of the RSF model and the Cox model after linear relationship assessment.** (A) RSF model and the Cox model in the internal validation set; (B) RSF model and the Cox model in the external validation set.

Abbreviations: Cox: Cox proportional hazards; RSF: random survival forest.

**
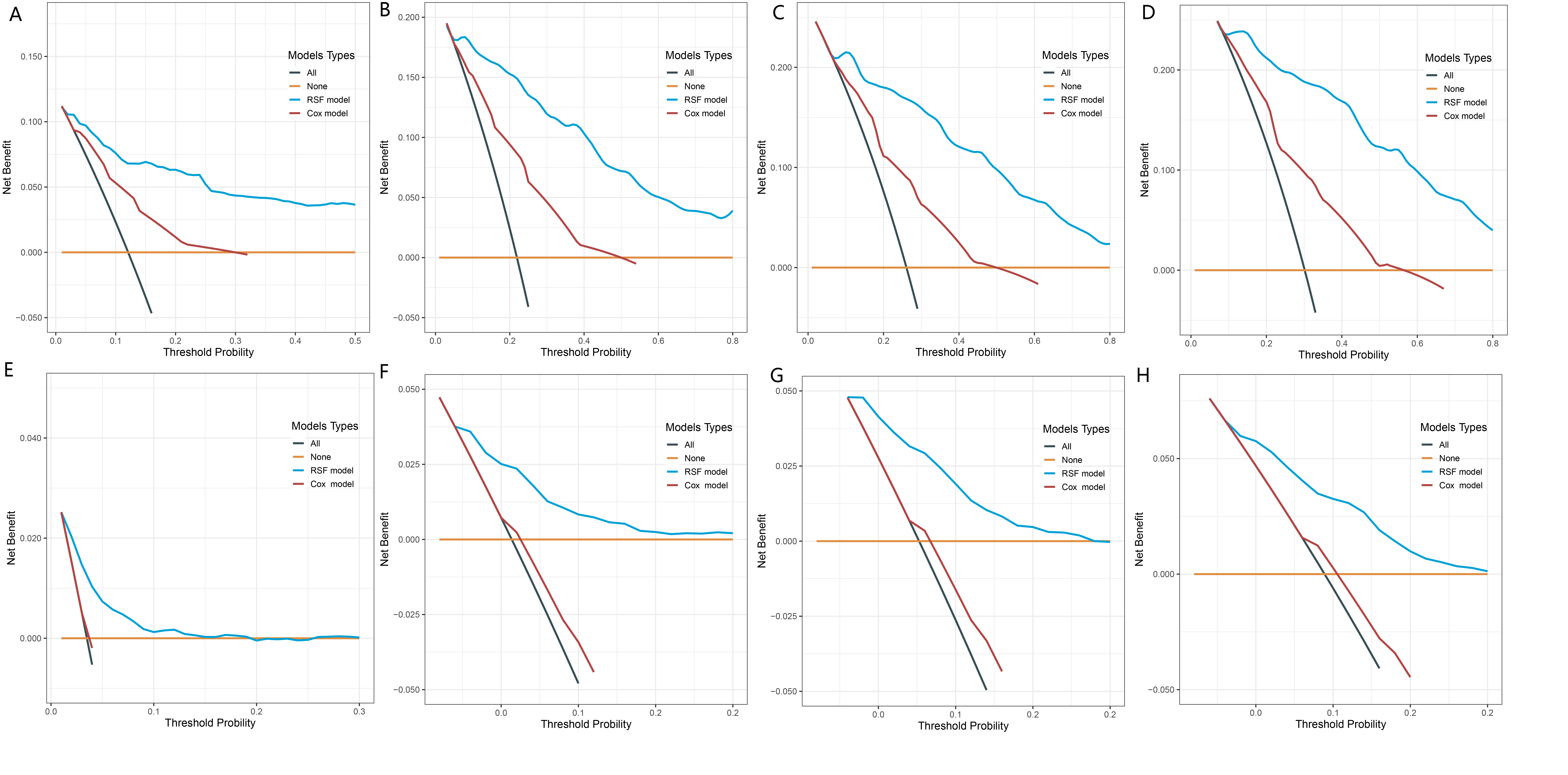
**

**DCA curves of the RSF model and the Cox model after linear relationship assessment.** (A, B, C, D) RSF model and the Cox model after linear relationship assessment in the internal validation set in 1, 3, 5, 8 years, respectively; (E, F, G, H) RSF model and the Cox model after linear relationship assessment in the external validation set in 1, 3, 5, 8 years, respectively.

Abbreviation: Cox: Cox proportional hazards; DCA: decision curve analysis; RSF: random survival forest.

**NRIs of the RSF model and the Cox model after linear relationship assessment in the internal and external validation sets**

| **Time** | **NRI** | |
| --- | --- | --- |
|  | **Internal validation set** | **External validation set** |
| 1-year | 0.379 (0.280 - 0.487) | 0.007 (-0.001 - 0.070) |
| 2-year | 0.498 (0.431 - 0.585) | 0.025 (-0.000 - 0.085) |
| 3-year | 0.470 (0.411 - 0.542) | 0.050 (0.011 - 0.127) |
| 4-year | 0.492 (0.418 - 0.557) | 0.072 (0.013 - 0.113) |
| 5-year | 0.544 (0.446 - 0.614) | 0.072 (0.040 - 0.129) |
| 6-year | 0.530 (0.425 - 0.580) | 0.079 (0.042 - 0.121) |
| 7-year | 0.559 (0.454 - 0.640) | 0.076 (0.032 - 0.116) |
| 8-year | 0.555 (0.388 - 0.677) | 0.069 (0.024 - 0.131) |

Abbreviations: NRI: net reclassification improvement
