# Supplementary material for: Development and Spatial External Validation of a Predictive Model of Survival Based on Random Survival Forest Analysis for People Living With HIV and AIDS After Highly Active Antiretroviral Therapy in China: Retrospective Cohort Study
Source: J Med Internet Res. 2025 Jun 2;27:e71257. doi: 10.2196/71257 (PMC12171649; doi:10.2196/71257)
Supplement: Multimedia Appendix 12 [file jmir_v27i1e71257_app12.docx]

**Multimedia Appendix 12. Sensitivity analysis 4: The performance of the RSF models by age groups in the internal and external validation sets**

| **Subgroups** | **Internal validation set** | | **External validation set** | |
| --- | --- | --- | --- | --- |
|  | **C index (95% CI)** | **iAUC (95% CI)** | **C index (95% CI)** | **iAUC (95% CI)** |
| ＜ 60 years | 0.924 (0.906 - 0.942) | 0.910 (0.889 - 0.932) | 0.746 (0.715 - 0.778) | 0.746 (0.715 - 0.778) |
| ≥ 60 years | 0.815 (0.749 - 0.881) | 0.772 (0.700 - 0.846) | 0.635 (0.584 - 0.686) | 0.620 (0.560 - 0.680) |

Abbreviations: C index: consistency index; iAUC: integrated area under curve
